# Nano‐Brake Halts Mitochondrial Dysfunction Cascade to Alleviate Neuropathology and Rescue Alzheimer's Cognitive Deficits

**DOI:** 10.1002/advs.202204596

**Published:** 2023-01-26

**Authors:** Qian Zhang, Qingxiang Song, Renhe Yu, Antian Wang, Gan Jiang, Yukun Huang, Jun Chen, Jianrong Xu, Dayuan Wang, Hongzhuan Chen, Xiaoling Gao

**Affiliations:** ^1^ Department of Pharmacology and Chemical Biology State Key Laboratory of Oncogenes and Related Genes Shanghai Universities Collaborative Innovation Center for Translational Medicine Shanghai Jiao Tong University School of Medicine 280 South Chongqing Road Shanghai 200025 China; ^2^ School of Pharmacy Shanghai Pudong Hospital & Department of Pharmaceutics Fudan University Lane 826, Zhangheng Road Shanghai 201203 China; ^3^ Academy of Integrative Medicine Shanghai University of Traditional Chinese Medicine 1200 Cailun Road Shanghai 201203 China; ^4^ Institute of Interdisciplinary Integrative Biomedical Research Shuguang Hospital Shanghai University of Traditional Chinese Medicine 1200 Cailun Road Shanghai 201203 China

**Keywords:** Alzheimer's disease, Ca^2+^ homeostasis, mitochondrial dysfunction, mitochondrial permeability transition pore, nanotherapeutics

## Abstract

Mitochondrial dysfunction has been recognized as the key pathogenesis of most neurodegenerative diseases including Alzheimer's disease (AD). The dysregulation of mitochondrial calcium ion (Ca^2+^) homeostasis and the mitochondrial permeability transition pore (mPTP), is a critical upstream signaling pathway that contributes to the mitochondrial dysfunction cascade in AD pathogenesis. Herein, a “two‐hit braking” therapeutic strategy to synergistically halt mitochondrial Ca^2+^ overload and mPTP opening to put the mitochondrial dysfunction cascade on a brake is proposed. To achieve this goal, magnesium ion (Mg^2+^), a natural Ca^2+^ antagonist, and siRNA to the central mPTP regulator cyclophilin D (CypD), are co‐encapsulated into the designed nano‐brake; A matrix metalloproteinase 9 (MMP9) activatable cell‐penetrating peptide (MAP) is anchored on the surface of nano‐brake to overcome the blood‐brain barrier (BBB) and realize targeted delivery to the mitochondrial dysfunction cells of the brain. Nano‐brake treatment efficiently halts the mitochondrial dysfunction cascade in the cerebrovascular endothelial cells, neurons, and microglia and powerfully alleviates AD neuropathology and rescues cognitive deficits. These findings collectively demonstrate the potential of advanced design of nanotherapeutics to halt the key upstream signaling pathways of mitochondrial dysfunction to provide a powerful strategy for AD modifying therapy.

## Graphical abstract

Matrix metalloproteinase 9 activatable cell‐penetrating peptide (MAP) on the nano‐brake mediates its efficient entry into the target cells. With siRNA reducing the expression of cyclophilin D and magnesium ion suppressing calcium ion overload, nano‐brake halts the overall mitochondrial dysfunction cascade in the cerebrovascular endothelial cells, neurons, astrocytes and microglia and largely alleviates the neuropathology and cognitive deficits of Alzheimer's disease.

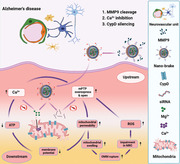

## 1 Introduction

During the aging process, mitochondria decrease in both quality and functionality.^[^ ^1^, ^2^, ^3^ ^]^ Mitochondrial dysfunction has been recognized as the primary pathogenesis of almost all neurodegenerative diseases, such as Alzheimer's disease (AD), the most common type of dementia.^[^ ^2^, ^4^ ^]^ Impaired mitochondrial morphology and function, imbalance of mitochondrial dynamics, and redistribution of mitochondria have been observed in various AD brain cell types including the cerebrovascular endothelial cells, neurons, and glial cells.^[^ ^3^, ^5^, ^6^, ^7^, ^8^, ^9^, ^10^, ^11^ ^]^ Sustained mitochondrial damage leads to the opening and overexpression of mitochondrial permeability transition pore (mPTP), leakage of cytochrome C into the cytoplasm, decreased production of adenosine triphosphate (ATP), and increased burden of reactive oxygen species (ROS), leading to brain cell dysfunction and even cell death.^[^ ^3^, ^12^ ^]^ Therefore, therapeutics that can halt mitochondrial damage and improve mitochondrial function will provide a promising solution for the management of AD.^[^ ^13^, ^14^ ^]^

In order to treat AD through the modulation of mitochondrial function, various nanotherapeutics such as cerium oxide nanoparticles, gold nanoparticles, and red blood cell membrane‐coated nanostructure have been developed.^[^ ^15^, ^16^, ^17^, ^18^ ^]^ However, most of the previous approaches target the ROS signaling pathway, the downstream process of mitochondrial damage, and can't entirely halt the mitochondrial damage cascades. Therapeutics that halt the key upstream signaling pathways of mitochondrial dysfunction are still unavailable.^[^ ^19^ ^]^

In the pathogenesis of AD, dysregulated mitochondrial calcium ion (Ca^2+^) homeostasis plays a central role. Misfolded amyloid beta (A*β*) and hyperphosphorylated tau disrupt mitochondrial Ca^2+^ homeostasis in various brain cells, while the excessive level of Ca^2+^ acts as the principal trigger of mitochondrial dysfunction by enhancing the production of ROS, reducing the production of ATP and damaging the permeabilization of the mitochondrial membrane. mPTP, a high‐conductance channel, is well regulated by cyclophilin D (CypD) to maintain mitochondrial Ca^2+^ homeostasis. A*β* aggregates and other neurotoxic agents interact with CypD to induce mitochondrial depolarization through mPTP activation due to mitochondrial ROS and Ca^2+^ overload. Once the above processes cannot be put on brake, mitochondrial cascade dysfunction and cell damage are inevitable.^[^ ^1^, ^10^, ^20^, ^21^ ^]^ Accordingly, developing an approach to preserve Ca^2+^ balance and mitochondrial homeostasis is crucial for avoiding or alleviating the underlying pathology in AD.

To test such hypothesis, here we propose a “two‐hit braking” therapeutic strategy to synergistically halt mitochondrial Ca^2+^ overload and mPTP opening to put the mitochondrial dysfunction cascade on a brake. Magnesium (Mg^2+^), which can act as a naturally powerful Ca^2+^ antagonist due to its similar atomic structure to Ca^2+^, was incorporated to conquer the hit one, Ca^2+^ overload; siRNA to CypD, the central mPTP regulator, was applied to halt mPTP opening, the hit two.^[^ ^22^ ^]^ What is more, Mg^2+^ could naturally form nano‐precipitate with CypD siRNA to realize simultaneous encapsulation of Mg^2+^ and CypD siRNA into the core of a liposome (Mg‐CypD‐LNC). To overcome the blood‐brain barrier (BBB) and realize targeted delivery to the lesion sites, Mg‐CypD‐LNC was anchored with a matrix metalloproteinase 9 (MMP9) activatable cell‐penetrating peptide (MAP) on its surface to obtain the nano‐brake. After entering the blood circulation, MAP on nano‐brake would be specifically cleaved by the overexpressed MMP9 at the damaged cerebrovascular sites in AD, and then present the positive‐charged arginine‐rich cell‐penetrating peptide to mediate efficient delivery of nano‐brake into the target cells and get access to the mitochondria.^[^ ^23^ ^]^ With siRNA reducing the CypD expression and Mg^2+^ suppressing the Ca^2+^ overload, the overall mitochondrial dysfunction cascade event in the cerebrovascular endothelial cells, neurons, and microglia will be put on brake and the AD neuropathology and cognitive deficits are expected to be largely relieved.

## 2 Results and Discussion

### 2.1 Preparation and Characterization of Nano‐Brake

**Figure 1:**
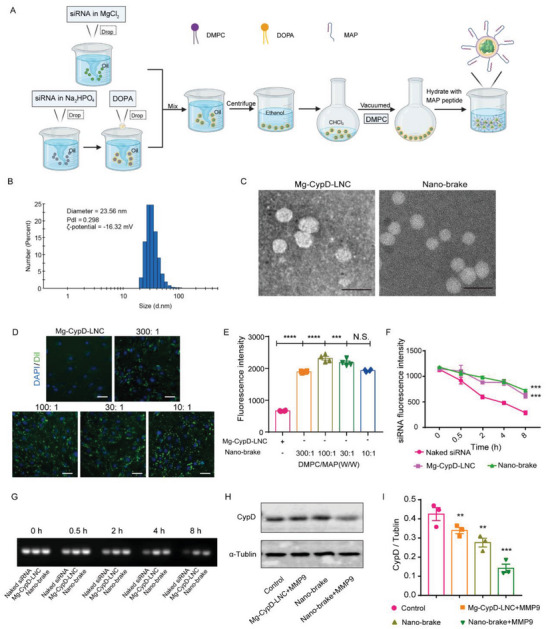
Construction and characterization of nano‐brake. A) Reversed‐phase microemulsion followed by thin‐film hydration was used to prepare nano‐brake. B) The size distribution of nano‐brake as detected by dynamic light scattering (DLS). C) Morphology of Mg‐CypD‐LNC and nano‐brake under transmission electron microscopy (TEM). Scale bar, 50 nm. D,E) bEnd.3 cells were incubated with DiI‐labeled nano‐brake at the DMPC to MAP weight ratio 300:1, 100:1, 30:1, and 10:1, and Mg‐CypD‐LNC for 3 h, D) Fluorescence images and E) intracellular fluorescence quantitative results. Data represent the mean ± SEM. ***p < 0.001, ****p < 0.0001, significantly different compared with that of Mg‐CypD‐LNC. N.S., no significant difference compared with 30:1, *n* = 4. Scale bar, 25 µm. F,G) Nano‐brake protects siRNA from degradation. After incubation in 10% fetal bovine serum, the remaining siRNA content in nano‐brake, Mg‐CypD‐LNC, and naked siRNA was determined at different time points. Data represent the mean ± SEM. ***p < 0.001, significantly different compared with that of 8 h naked siRNA, *n* = 3. H,I) bEnd.3 cells were incubated with DMEM, Mg‐CypD‐LNC (pre‐incubating with MMP9 protein), nano‐brake, and nano‐brake (pre‐incubating with MMP9 protein), respectively, at a concentration of 100 nM siRNA for 48 h. H) CypD expression was determined by western blot. I) Statistical chart of CypD expression in bEnd.3 cells. Data represent the mean ± SEM. **p < 0.01, ***p < 0.001, significantly different compared with that of control, *n* = 3.

To construct the nano‐brake, we first prepared a siRNA‐loaded magnesium phosphate core (Mg‐siRNA core) via the water‐in‐oil microemulsion method (**Figure** **1**A). Magnesium phosphates are of good biodegradability and high biocompatibility as they are a key component of human hard tissues.^[^ ^24^, ^25^ ^]^ Magnesium ions can also form complexes with the nucleic acid backbone to protect the double‐stranded siRNA products from serum nucleases attack.^[^ ^26^ ^]^ 1,2‐dioleoyl‐sn‐glycero‐3‐phosphate acid (DOPA) was then applicated to coat the surface of the Mg‐siRNA core, which was subsequently encapsulated by 1,2‐dimyristoyl‐sn‐glycerol‐3‐phosphocholine (DMPC). The resulting nanoparticles loaded with CypD siRNA and negative control (NC) siRNA were named CypD siRNA‐loaded lipid nanocarrier (Mg‐CypD‐LNC) and NC siRNA‐loaded lipid nanocarrier (Mg‐LNC), respectively. Previous reports indicated that the molar ratio of Ca^2+^ to H*_x_* PO_4_ *^x^* ^‐3^ (Ca/P) may lead to precipitation with different particle sizes and various zeta potential.^[^ ^27^ ^]^ Accordingly, here we explored the effect of Mg^2+^ to H*_x_* PO_4_ *^x^* ^‐3^ (Mg/P) molar ratio on zeta potential, particle size, siRNA encapsulation efficiency, and the Mg^2+^ loading capacity. It was found that the particle size of Mg‐LNC ranged from 20.16 to 47.21 nm at the Mg/P molar ratio from 8 to 200 (Figure S1A, Supporting Information). Zeta potential reached the peak (−22.7 ± 2.0 mV) at the Mg/P ratio from 8 to 15 (Figure S1B, Supporting Information). The highest siRNA encapsulation efficiency (63.68 ± 0.37%) was achieved at the Mg/P ratio 15 (Figure S1C, Supporting Information). In contrast, the amount of Mg^2+^ precipitated gradually increased with the increase of the Mg/P ratio and achieved the maximum Mg^2+^ loading (4.45 ± 0.21 mM in 1 mg mL^−1^ DMPC LNC) at the Mg/P ratio 200 (Figure S1D, Supporting Information). Transmission electron microscopy (TEM) analysis showed that at the Mg/P ratio 8, 12.5, 25, and 50, the Mg‐siRNA core exhibited good dispersibility, spherical shapes, and uniform particle size (Figure S1E, Supporting Information). Taking all the above factors into consideration, especially, for the balance of encapsulation efficiency of siRNA and the amount of Mg^2+^ in the carrier, both of which work together to repair mitochondrial dysfunction, Mg/P ratio 25 was selected to prepare Mg‐CypD‐LNC and Mg‐LNC.

After that, we incorporated MAP into the lipid membrane of Mg‐CypD‐LNC and Mg‐LNC by fusing MAP with an *α*‐helix peptide which mimics the lipid‐binding motif of apolipoprotein A‐I to obtain the nano‐brake and MAP‐Mg‐LNC, respectively. According to TEM and dynamic light scattering (DLS) measurements, the diameter of the nano‐brake was ≈ 25 nm and displayed a regular spherical shape (Figure 1B,C). The zeta potential of the nano‐brake was around −16 mV. The encapsulation efficiency of siRNA was 56.5 ± 2.1% and Mg^2+^ concentration was 1.79 mM (in 1 mg mL^−1^ DMPC LNC). The cellular internalization of DiI‐nano‐brake at different densities of MAP peptide was determined in a mouse brain microvascular endothelial cell line bEnd.3 cells. It was found that the incorporation of MAP significantly improved the cellular uptake of the nanoformulation, and the highest cellular uptake of nano‐brake was achieved at the DMPC to MAP weight ratio of 100 to 1. We extrapolated that the highest cellular uptake achieved at this ratio could be mainly owing to the best exposure of MAP to MMP9 chopping (Figure 1D,E, and Figure S2, Supporting Information). Next, we determined the profiles of siRNA release from nano‐brake and lysosome. It was found that most of the siRNA release from DiD‐nano‐brake (pretreated with MMP9) after 3 h incubation and the co‐localization between siRNA (green) and lysosome (red) was kept at a low level (co‐localization index always lower than 7.4% ± 0.4%) from 0.5 to 3 h. These results suggested that siRNA was successfully released from nano‐brake to the cytoplasm (Figures S3 and S4, Supporting Information). Furthermore, we performed a CCK8 assay to evaluate the toxicity of nano‐brake in bEnd.3 and SH‐SY5Y cells. For the analysis, the cells were treated with nano‐brake at the siRNA concentration ranging from 25 to 300 nM for 24 h. The viability of bEnd.3, SH‐SY5Y was higher than 90% even when the concentration of siRNA was up to 300 nM (Figure S5, Supporting Information), suggesting that nano‐brake exhibited no significant cytotoxicity.

The serum stability of siRNA loaded in nano‐brake was tested using 10% fetal bovine serum to assess the capacity of nano‐brake as a carrier for intracellular siRNA delivery. It was found that 78.3% of naked siRNA was degraded at 8 h, while 38.7% of siRNA in nano‐brake and 45.6% of siRNA in Mg‐CypD‐LNC were degraded under the same condition (Figure 1F,G). The storage stability was also determined, finding that the size and zeta potential of the nano‐brake exhibited negligible changes and the siRNA in nano‐brake was stable for 14 days (Figure S6, Supporting Information). Such findings suggested that the encapsulation of siRNA in nano‐brake and Mg‐CypD‐LNC could efficiently protect siRNA from degradation.

After that, we performed a western blot to evaluate the effect of the nano‐brake on CypD expression, finding that nano‐brake effectively reduced the expression of CypD in bEnd.3 cells in the presence of MMP9 protein. Compared with the DMEM control, Mg‐CypD‐LNC treatment decreased the expression of CypD by 19.9%. In the absence of MMP9, nano‐brake silenced the expression of CypD by 34.9%, while in the presence of MMP9, nano‐brake silenced the expression of CypD by 66.8% (Figure 1H,I). Moreover, after treatment with nano‐brake, the expression of CypD in BV2 and SH‐SY5Y cells was decreased by 50.8% and 35.0%, respectively (Figure S7, Supporting Information). These results indicated that siRNA carried by nano‐brake could effectively enter the target cells and exert their gene knockdown function.

### 2.2 Nano‐Brake Efficiently Targeted the Damaged Cerebral Microvasculature and Achieved Intracerebral Delivery in AD Model Mice

**Figure 2:**
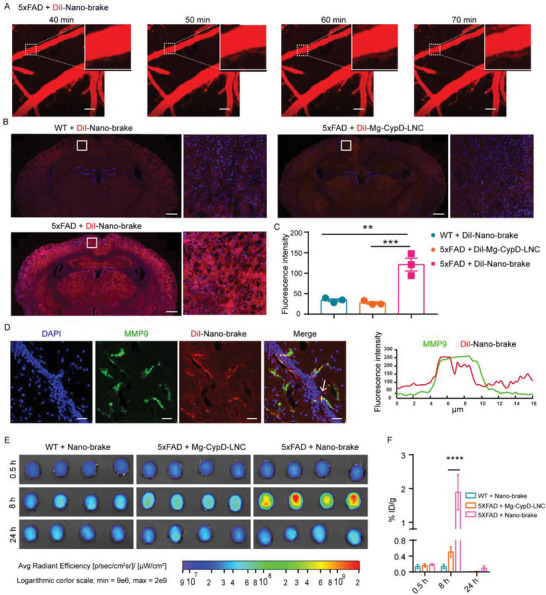
Nano‐brake efficiently targeted the damaged cerebral microvasculature to achieve intracerebral delivery in AD model mice. A–D) DiI‐labeled nano‐brake and Mg‐CypD‐LNC (41.3 µg kg^−1^ siRNA) were administrated through the tail vein of 6‐month‐old 5xFAD mice and WT mice. A. Two‐photon imaging images of nano‐brake distributed around the cerebral vessels of 5xFAD mice. Scale bar, 40 µm. B) The distribution of nano‐brake and Mg‐CypD‐LNC in the brain slices of 5xFAD mice and WT mice were observed using a laser confocal microscope, Scale bar, 150 µm. C) The fluorescence intensity of nano‐brake and Mg‐CypD‐LNC in the brain slices of 5xFAD mice and WT mice, respectively, *n* = 3, **p < 0.01, ***p < 0.001, significantly different compared with that of diI‐nano‐brake treated 5xFAD group. D) Left, DiI‐nano‐brake colocalized with MMP9 in brain slice of those 5xFAD mice treated with DiI‐nano‐brake, scale bar, 40 µm. Right, the signal intensity profile of MMP9 (green line) and DiI‐nano‐brake (red line) at the position labeled with a yellow line in the merged image (left, pointed by white arrow). E) DiR‐nano‐brake achieved higher brain entry efficiency in 5xFAD mice. Six‐month‐old 5xFAD mice and littermate WT mice were injected with DiR‐labeled nano‐brake or Mg‐CypD‐LNC (82.5 µg kg^−1^ siRNA) via the tail vein. F) The brain distribution of d9‐nano‐brake and d9‐Mg‐CypD‐LNC in 5xFAD mice and WT mice at 8 h after administration and analyzed by LC‐MS/MS, displaying as % ID/g, respectively, *n* = 4. Data represent the mean ± SEM. ****p < 0.0001, significantly different compared with that of 8 h Mg‐CypD‐LNC treated 5xFAD group.

As indicated previously, MMP9 expression was increased in the injured cerebral endothelial cells in 5xFAD mice, which overexpress mutant human amyloid beta with five mutations and exhibit amyloid plaque pathology similar to that found in AD patients (Figure S8, Supporting Information).^[^ ^28^ ^]^ To see whether nano‐brake can target the injured cerebral microvasculature efficiently, DiI‐nano‐brake and DiI‐Mg‐CypD‐LNC were injected into 5xFAD mice and the wild‐type control animals (WT) via the caudal vein. Under two‐photon microscopy, it was found that in 5xFAD mice, DiI‐nano‐brake achieved much higher adhesion along and permeation across the cerebral vessels than DiI‐Mg‐CypD‐LNC (**Figure** **2**A, Video S1 and Figure S9, Supporting Information). Specifically, at 4 h after administration, compared with that of DiI‐Mg‐CypD‐LNC in the brains of 5xFAD mice and DiI‐nano‐brake in the brains of WT mice, the fluorescence intensity of DiI‐nano‐brake in the brains of 5xFAD mice was enhanced by 3.17 and 2.57 times, respectively (Figure 2B,C). Laser confocal microscopy analysis also witnessed a higher level of DiI‐nano‐brake accumulating along the microvascular walls than that in the parenchyma (Figure 2B,D), indicating that before entering the parenchyma, DiI‐nano‐brake first enriched in the damaged blood vessels of AD. Moreover, DiI‐nano‐brake was also found well colocalized with MMP9, exhibiting a typical cerebral capillary morphological profile and a relatively weak accumulation in other cells in the brain (Figure 2D). Compared with that in the brains of WT mice, nano‐brake achieved much higher accumulation in the brain of 5xFAD mice, which was also much higher than that of Mg‐CypD‐LNC in 5xFAD mice (Figure 2E and Figure S10, Supporting Information). Consistently, LC‐MS/MS quantitative analysis demonstrated that in 5xFAD mice, the level of deuterium isotope‐labeled DMPC (d9‐DMPC) detected in the brains of 5xFAD mice at 8 h after administration of d9‐labeled nano‐brake was 3.75 times higher than that following the treatment of d9‐labeled Mg‐CypD‐LNC (Figure 2F). Additionally, DiR‐nano‐brake showed similar distribution profiles in the peripheral organs of 5xFAD mice with Mg‐CypD‐LNC (Figure S11, Supporting Information), while the limited MMP9 expression in normal organs and the MMP9‐responsive design of nano‐brake would considerably reduce its off‐target effect.^[^ ^29^ ^]^ These results clearly demonstrated that MAP can effectively promote the intracerebral delivery of nano‐brake in 5xFAD mice.

### 2.3 Nano‐Brake Alleviated A*β*‐Induced Mitochondria Impairment In Vitro

**Figure 3:**
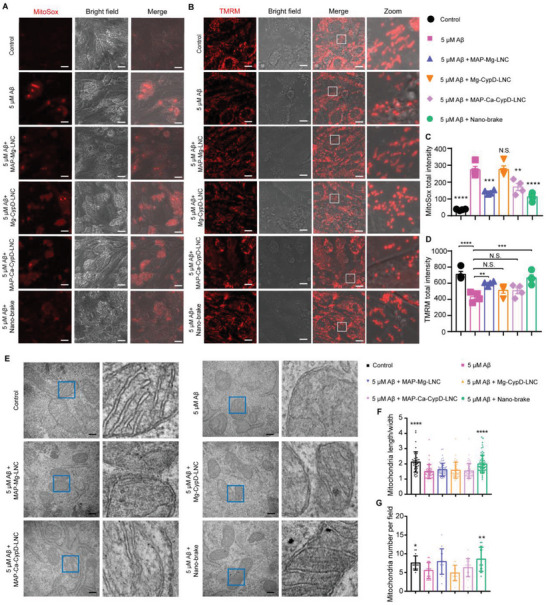
Nano‐brake alleviated A*β*‐induced mitochondria impairment in vitro. bEnd.3 cells were co‐incubated with 5 µM A*β*_1‐42_ oligomer and the formulations (Mg‐CypD‐LNC, MAP‐Mg‐LNC, MAP‐Ca‐CypD‐LNC, or nano‐brake) for 48 h. A) Nano‐brake alleviated A*β*_1‐42_‐induced mitochondrial superoxide production (indicated by MitoSox, red). Scale bar, 20 µm. B) Nano‐brake alleviates A*β*_1‐42_‐induced mitochondrial membrane potential decrease (indicated by TMRM, red). Scale bar, 20 µm. C) Quantitative analysis of nano‐brake alleviation of A*β*_1‐42_ induced mitochondrial superoxide production. Data represent the mean ± SD, *n* = 4,5. **p < 0.01, ***p < 0.001, ****p < 0.0001, significantly different with that of the A*β*_1‐42_ treatment group, n.s., not significant, compared to A*β*_1‐42_ treatment group. D) Quantitative analysis of nano‐brake alleviation of A*β*_1‐42_ induced mitochondrial membrane potential decrease. Data represent the mean ± SD, *n* = 4,5. **p < 0.01, ***p < 0.001, ****p < 0.0001, significantly different from that of the A*β* group, n.s., not significant, compared to the A*β*_1‐42_ treatment group. E) TEM analysis of nano‐brake alleviation of A*β*_1‐42_‐induced mitochondrial damage. Scale bar, 200 nm. F) Statistical analysis of mitochondria length/width ratio in the different treatment groups. Data represent the mean ± SD, *n* = 42–68 mitochondria. ****p < 0.0001, significantly different from that of the A*β*_1‐42_ treatment group. G) Statistical analysis of the number of mitochondria in the different treatment groups. Data represent the mean ± SD, *n* = 13–17 fields. *p < 0.05, **p < 0.01, significantly different with that of the A*β*_1‐42_ treatment group.

It has been shown that A*β* can induce mitochondrial damage by causing cytosolic and mitochondrial Ca^2+^ overload in various AD brain cell types.^[^ ^30^, ^31^ ^]^ Therefore, exposure bEnd.3, SH‐SY5Y and BV2 cells to A*β*_1‐42_ oligomer could evoke mitochondrial damage, which was used to assess the mitochondrial repairment of nano‐brake (Figures S12 and S13, Supporting Information). To illustrate the mitochondrial protective effect of Mg^2+^ and assess its advantages as a siRNA carrier for the management of neurodegenerative diseases, we took MAP‐Ca‐CypD‐LNC (with calcium phosphate core to carry siRNA) as the control group (Figure S14, Supporting Information).^[^ ^32^ ^]^ To replicate MAP activation by the overexpressed MMP9 at injured cerebrovascular regions in vivo, nano‐brake, MAP‐Mg‐LNC, Mg‐CypD‐LNC, and MAP‐Ca‐CypD‐LNC were pretreated with MMP9 for 2 h. After that, the cells were co‐incubated with A*β*_1‐42_ oligomer and nano‐brake, MAP‐Mg‐LNC, Mg‐CypD‐LNC, and MAP‐Ca‐CypD‐LNC for 48 h, respectively. It was found that the A*β*_1‐42_ oligomer significantly enhanced mitochondrial ROS (indicated by the increased MitoSOX intensity) and reduced mitochondrial membrane potential (indicated by the decreased fluorescence intensity of TMRM) (**Figure** **3**A–D). Mg‐CypD‐LNC treatment failed to reverse the A*β*_1‐42_ oligomer‐induced mitochondrial damage, while MAP‐Mg‐LNC and MAP‐Ca‐CypD‐LNC slightly relieved the mitochondrial damage. Strikingly, nano‐brake recovered the mitochondrial ROS and mitochondrial membrane potential (decreased by 59.0% and increased by 54.4%, respectively, compared with the A*β*_1‐42_‐treated group) (Figure 3A–D). In the neuroblastoma cell line SH‐SY5Y cells and mouse microglial cell line BV2 cells, nano‐brake also largely restored the A*β*_1‐42_ oligomer‐induced changes of the mitochondrial membrane potential and mitochondrial ROS (Figures S15 and S16, Supporting Information).

The mitochondrial bioenergetic function closely relates to mitochondrial morphology and density.^[^ ^33^ ^]^ Accordingly, we characterized the mitochondrial morphology and density in bEnd.3 cells through TEM following the incubation with A*β*_1‐42_ oligomer, it was found that the mitochondria become swollen, shortened, and fragmented, and their density decreases. MAP‐Mg‐LNC, Mg‐CypD‐LNC and MAP‐Ca‐CypD‐LNC all failed to reverse the above mitochondrial abnormalities, while nano‐brake efficiently alleviated the A*β*‐induced mitochondria impairment (Figure 3E–G), indicating that MAP‐medicated intracellular accumulation, as well as the combination between Mg and CypD siRNA, are all important to the successful mitochondrial protection.

### 2.4 Nano‐Brake Attenuated Mitochondria Impairment in the Brain of AD Model Mice

**Figure 4:**
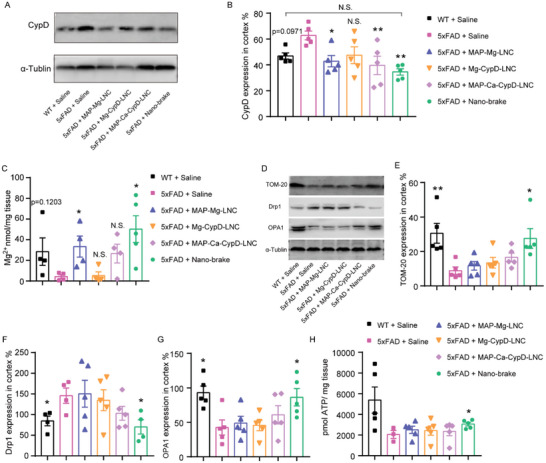
Nano‐brake attenuated mitochondria impairment in AD model mice. Six‐month‐old 5xFAD mice were treated with nano‐brake, MAP‐Mg‐LNC, Mg‐CypD‐LNC, or MAP‐Ca‐CypD‐LNC (82.5 µg kg^−1^ siRNA) for 4 weeks. WT mice and 5xFAD mice treated with saline served as the normal and negative controls, respectively. A) Western blot analysis of CypD levels in the mice cortex. B) Quantitative analysis of CypD expression in the mice cortex. Data represent the mean ± SD, *n* = 5. n.s. showed no significant difference between the two groups, *p < 0.05, **p < 0.01, significantly different from that of the 5xFAD mice treated with saline group. C) Mg^2+^ levels in the cortex of mice. Data represent the mean ± SD, *n* = 5. D) Western blot results of TOM‐20, Drp1, and OPA1 in the cortex of mice in different treatment groups. D–G) Statistical chart of TOM‐20, Drp1, and OPA1 expression in the cortex of mice. Data represent the mean ± SEM, *n* = 4,5. H. ATP levels in the mice cortex. Data represent the mean ± SD, *n* = 5. C,E–H) *p < 0.05, **p < 0.01, significantly different with that of 5xFAD mice treated with saline group.

Mitochondrial dysfunction is an early pathological feature in AD.^[^ ^33^ ^]^ As the mitochondrial mPTP overexpression and Ca^2+^ overloading play a central role in AD mitochondrial cascade dysfunction, we continued to determine whether nano‐brake can restore the mitochondrial dysfunction in vivo in AD model animals. Compared with that in the saline‐treated WT mice, the expression of CypD in the saline‐treated 5xFAD mice was 33.9% higher, confirming that CypD is abnormally increased in the pathological state of AD. After the nano‐brake, MAP‐Mg‐LNC, and MAP‐Ca‐CypD‐LNC treatment, CypD expression significantly decreased. Specifically, nano‐brake decreased CypD expression by 44.9% (compared with that of saline‐treated 5xFAD mice), reaching the level of CypD expression in the saline‐treated WT mice, obviously superior to other formulations (MAP‐Mg‐LNC by 31.8% and MAP‐Ca‐CypD‐LNC by 36.9%, respectively). Interestingly, MAP‐Mg‐LNC also reduced CypD expression, indicating that Mg^2+^ has the ability to regulate CypD expression, possibly by acting as a Ca^2+^ antagonist and inhibiting mPTP opening^[^ ^36^ ^]^ (**Figure** **4**A,B).

The Mg^2+^ concentration in brain tissue was determined to assess the ability of the nano‐brake to transport Mg^2+^ to the brain. Mg^2+^ levels in 5xFAD mice were found to be lower than that in the WT mice. No significant difference in Mg^2+^ concentration in the brain was found among the Mg‐CypD‐LNC, MAP‐Ca‐CypD‐LNC, and the saline‐treated 5xFAD control. In contrast, in the MAP‐Mg‐LNC and nano‐brake‐treated mice, Mg^2+^ concentration was increased close to that in the WT animals, indicating that both MAP‐Mg‐LNC and nano‐brake successfully delivered Mg^2+^ into the brain (Figure 4C). The increased Mg^2+^ would modulate mitochondrial functions directly by acting as a Ca^2+^ antagonist.

We further assess the effect of nano‐brake on mitochondrial functional protein levels (Figure 4D–G). The expression of mitochondrial outer membrane 20 (TOM‐20) was shown to be considerably decreased in Alzheimer's disease, which was closely linked to mitochondrial dysfunction .^[^ ^34^ ^]^ We discovered that, compared to that in the WT mice, the expression of TOM‐20 in 5xFAD mice was reduced by 70.0%, but was totally restored after treatment with nano‐brake (Figure 4E). Dynamin‐related protein 1 (Drp1) is a member of a highly conserved family of GTPase proteins and is essential for the division, size, and structure of mitochondria by being recruited onto the outer mitochondrial membrane to constrict mitochondria and drive fission.^[^ ^36^ ^]^ The expression of Drp1 in 5xFAD mice was 72.4% higher than that in the WT mice. Among all the nanoformulations, only nano‐brake significantly suppressed the expression of Drp1 (Figure 4F). Moreover, following the nano‐brake treatment in 5xFAD mice, the expression of optic atrophy 1 (OPA1), which is involved in mitochondrial fusion but decreased in AD,^[^ ^38^ ^]^ was increased by 102.7% (Figure 4G). Collectively, this evidence clearly demonstrated the effect of nano‐brake in recovering mitochondrial protein expression.

A proper level of Ca^2+^ in the mitochondrial matrix modulates oxidative phosphorylation activity and ATP production rate. In AD, not only A*β* but also other factors induce mitochondrial Ca^2+^ dyshomeostasis that impairs mitochondrial respiration, resulting in increased generation of ROS, decreased ATP synthesis, and altered mitochondrial membrane permeability, possibly leading to cell death. These chain reactions enhance the onset and progression of AD. Among all the nanoformulations, only nano‐brake significantly restored ATP production, indicating that MAP, Mg^2+^, and CypD siRNA, are essential for bioenergetic generation in mitochondria (Figure 4H).^[^ ^39^ ^]^

### 2.5 Nano‐Brake Ameliorated Neurovascular System Damage in AD Model Mice

**Figure 5:**
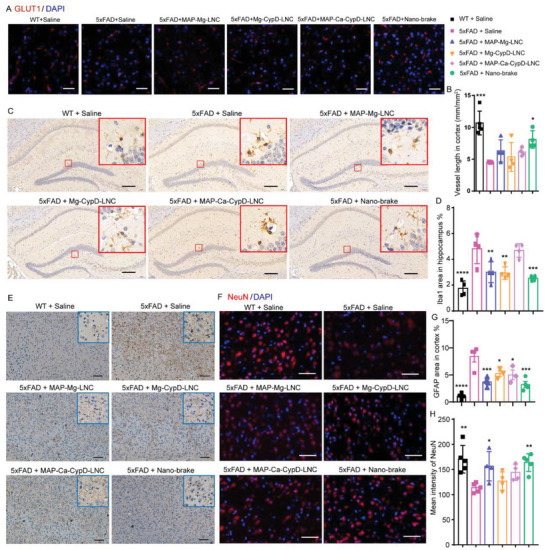
Nano‐brake alleviated damage of the neurovascular system in AD model mice. Six‐month‐old 5xFAD mice were treated with nano‐brake, MAP‐Mg‐LNC, Mg‐CypD‐LNC, and MAP‐Ca‐CypD‐LNC (82.5 µg kg^−1^ siRNA) for 4 weeks. WT mice and 5xFAD mice treated with saline served as the normal and negative controls, respectively. A) Immunofluorescence images of GLUT1 (red) on cerebral vessels of mice in different treatment groups, scale bar, 50 µm. B) Statistics of the length of cerebral vessels characterized by GLUT1 per unit area of mice in different treatment groups. Data represent the mean ± SD. *n* = 3,4. C) Immunohistochemistry images of Iba1 in the hippocampus of mice in different treatment groups, scale bar, 100 µm. D) Statistics of Iba1 positive area in the hippocampus of mice in different treatment groups. Data represent the mean ± SD. *n* = 4. E) The immunohistochemical images of GFAP in the cortex of mice in different treatment groups, scale bar, 100 µm. F) Immunofluorescence images of NeuN in the cortex of mice in different treatment groups, scale bar, 100 µm. G) Statistics of GFAP positive area in the cortex of mice. Data represent the mean ± SEM. *n* = 3,4. H. Immunofluorescence quantitative results of NeuN in the cortex of mice. Data represent the mean ± SD. *n* = 4,5. B,D,G,H) *p < 0.05, **p < 0.01, ***p < 0.001, ****p < 0.0001, significantly different with that of saline‐treated 5xFAD mice group.

Neurovascular impairment occurs early in AD and greatly contributes to irreversible neuronal function loss.^[^ ^40^ ^]^ All cell types in the neurovascular system, including endothelial cells, pericytes, astrocytes, microglia, and neurons, undergo pathological alterations in AD, with mitochondrial damage serving as a common mechanism. Therefore, we investigate whether mitochondrial recovery can successfully preserve neurovascular system cells. Cerebrovascular endothelial cells depend more on mitochondrial respiration than peripheral endothelial cells.^[^ ^40^ ^]^ In AD, mitochondrial damage in cerebrovascular endothelial cells causes an excessive generation of reactive oxygen species, which in turn produces cerebral endothelial apoptosis and capillary loss.^[^ ^9^ ^]^ By immunostaining cerebral capillaries with glucose transporter protein 1 (GLUT1), which is predominantly expressed in the cerebral vascular endothelium, we found that similar to the WT mice, the capillary length in the brains of nano‐brake‐treated animals was increased by 44.0% compared to the saline controls (**Figure** **5**A,B). This data demonstrated that nano‐brake successfully restored the cerebral vasculature.

Microglia are a key component of the neurovascular network. In neurodegenerative disorders, microglial mitochondria are defective, resulting in neuroinflammation and neuronal death.^[^ ^9^ ^]^ We also observed that microglia in 5xFAD mice exhibited an enlarged cell body and higher activation propensity (Iba1 area ratio of 4.8% ± 0.5%, relative to WT mice). After MAP‐Ca‐CypD‐LNC treatment, there was no discernible change in the area ratio of Iba1 in the hippocampus. MAP‐Mg‐LNC treatment slightly decreased microglia activation (3.0 ± 0.4%). In contrast, the nano‐brake treatment decreased microglia activation by 47.6% (Iba1 expression area ratio in the hippocampus was reduced to 2.5 ± 0.1%) (Figure 5C,D). Consistent with the reversed microglial activation, nano‐brake therapy considerably reduced neuroinflammation with IL‐6 and TNF‐*α* levels in the brain falling significantly (Figure S17, Supporting Information).

Mitochondrial damage in astrocytes also induces the activation of astrocytes.^[^ ^9^ ^]^ Glial fibrillary acidic protein (GFAP) immunostaining was used to assess the degree of astrogliosis. It was found that the GFAP expression in the cortex of 5xFAD mice was 8.4 ± 1.0%, while MAP‐Mg‐LNC treatment and nano‐brake treatment decreased GFAP expression to 3.7 ± 0.4% and 3.2 ± 0.5%, respectively (Figure 5E,G). This data demonstrated that nano‐brake can also efficiently relieve the activation of astrocytes.

Neurons are the primary component of the neurovascular network and the structural foundation of cognition. Damage to mitochondria is also seen as a critical early event before neuronal death.^[^ ^42^ ^]^ NeuN protein which is located in the nucleus and perinuclear cytoplasm of most neurons in the mammalian central nervous system was applied to reflect the health state of neurons.^[^ ^43^ ^]^ It was found that the intensity of NeuN fluorescence decreased by 33.3% in 5xFAD mice compared to WT animals, MAP‐Mg‐LNC treatment slightly restored NeuN levels, while nano‐brake treatment reversed NeuN levels to that of the WT group (Figure 5F,H).

Even though the control nanoformulations restored the endothelial cells, microglia, and astrocytes to varying degrees due to cell‐to‐cell heterogeneity, only nano‐brake can substantially restore each part of the neurovascular structure in the AD model mice, indicating that each component of nano‐brake, including MAP, Mg^2+^, and CypD siRNA, is essential for efficient mitochondrial protection.

The structural and functional changes in the neurovascular network contribute to disease pathology and cognitive decline in AD. In the “two‐hit braking” therapeutic strategy, CypD siRNA effectively silences the expression of CypD in the brains of AD model mice and repairs the dysregulation of neurovascular structure and function caused by mitochondrial damage from the downstream. Studies have shown that Mg^2+^ could also promote the clearance of A*β*.^[^ ^44^, ^45^ ^]^ Here, we found that the level of the A*β* plaques in the cortex of the MAP‐Ca‐CypD‐LNC‐treated animals was increased, suggesting that Ca^2+^ may promote A*β* deposition, which is in accord with previous studies.^[^ ^10^ ^]^ Interestingly, A*β* plaques in the hippocampus in 5xFAD mice decreased considerably following the treatment of nano‐brake and MAP‐Mg‐LNC. A*β* accumulation is one of the primary contributors to calcium ion imbalance and mitochondrial injury,^[^ ^46^ ^]^ in turn, Mg^2+^ and CypD siRNA may reduce mitochondrial damage by acting as Ca^2+^ antagonists and recovering mPTP to protect neurovascular function and promote A*β* clearance (Figure S18, supporting information).^[^ ^47^, ^48^ ^]^

### 2.6 Nano‐Brake Safely Rescued Cognitive Deficits in 5xFAD Mice

**Figure 6:**
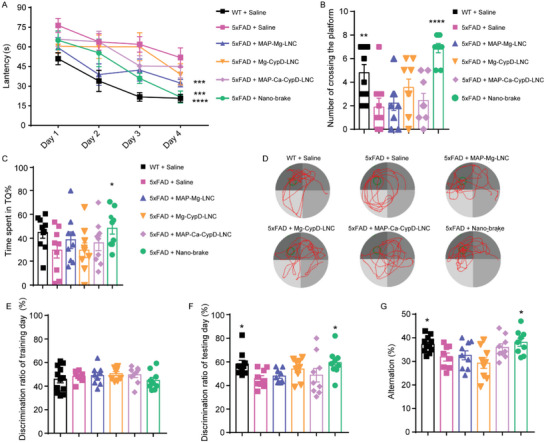
Nano‐brake rescued cognitive deficits in 5xFAD mice. Six‐month‐old 5xFAD mice were treated with nano‐brake, MAP‐Mg‐LNC, Mg‐CypD‐LNC, and MAP‐Ca‐CypD‐LNC (82.5 µg kg^−1^ siRNA) for 4 weeks. WT mice and 5xFAD mice treated with normal saline served as normal and negative controls, respectively. A) The latency of the Morris water maze navigation experiment. B) The number of crossing the platform in 90 s in the probe trial. C) The percentage of exploration time of the target platform quadrant (TQ%) in the probe trial. A–C) *n* = 9,10. D. The typical swimming trajectory of mice in each group in the probe trial. E) The preference of two identical objects on the training day of the novel object recognition (NOR) experiment, *n* = 9. F) The preference of different groups of mice for new objects on the testing day of the NOR experiment, *n* = 9. G) The spontaneous alternation rate of mice in the Y‐maze test was analyzed. *n* = 8,9. Data represent the mean ± SEM. *p < 0.05, **p < 0.01, ***p < 0.001, ****p < 0.0001, significantly different from that of saline‐treated 5xFAD mice group.

The Morris water maze (MWM) test was applicated to determine the mice's spatial memory. Compared to WT mice, 5xFAD animals treated with saline demonstrated significantly worse spatial learning and memory. The four‐week MAP‐Mg‐LNC therapy slightly lowered escape latency during the training trials, but the nano‐brake treatment significantly decreased escape latency, outperforming all other groups (**Figure** **6**A). Similarly, compared to saline treatment, MAP‐Mg‐LNC, Mg‐CypD‐LNC, and MAP‐Ca‐CypD‐LNC treatment did not significantly increase the number of platform crossings in the probe trial. In contrast, nano‐brake‐treated mice achieved a higher number of platform crossings (Figure 6B,D), and spent a significantly longer exploring time seeking in the target quadrant (Figure 6C,D). Furthermore, these phenotypes’ alleviation following nano‐brake treatment was not attributed to the change in swimming speed (Figure S19, Supporting Information). These data indicated that nano‐brake efficiently improves spatial learning and memory performance in 5xFAD mice.

Furthermore, the novel object recognition (NOR) and Y‐maze tests, which are behavioral tests for measuring rodents’ willingness to explore new objects and new environments, were used to determine the ability of spatial learning and memory in the AD model mice. In the NOR test, on the training day, the animals from the various groups showed no preference for the objects in the NOR test (Figure 6E). On the testing day, nano‐brake‐treated 5xFAD mice spent considerably more time investigating the novel object than the familiar object, with a greater discrimination ratio, when compared to saline‐treated 5xFAD controls (Figure 6F). Consistently, in the Y‐maze test, after four‐week treatment with nano‐brake, 5xFAD mice exhibited enhanced cognitive ability, comparable with WT mice (Figure 6G). Meanwhile, the cognitive improving ability of nano‐brake is more effective than that of other nanoformulation groups, indicating that the cleavable MAP modification further improved the AD treatment efficacy in vivo compared to the non‐MAP nanocarrier, and Mg^2+^, as well as CypD siRNA, are essential for protecting mitochondria and enhancing memory function in 5xFAD mice.

In addition, the safety of nano‐brake was evaluated, and there was no discernible difference in body weight change, blood biochemistry analysis of renal and liver damage marker, or hematoxylin‐and‐eosin (H&E) staining of key organs following a 4‐week therapy with nano‐brake (Figure S20, Supporting Information). These findings demonstrate that nano‐brake is biocompatible and has therapeutic application potential.

## 3 Conclusion

In summary, this study reported a novel nano‐system, nano‐brake, to realize simultaneous delivery of Mg^2+^ and CypD siRNA into the target cells to repair the mitochondrial dysfunction in AD. By effectively breaking the mitochondrial malfunctioning cascade in the brain, nano‐brake offered a viable strategy for avoiding or ameliorating the pathophysiology underlying AD. Both in vitro and in vivo results demonstrated that nano‐brake preferentially targeted the impaired cerebral microvasculature, attenuated mitochondria impairment, and largely alleviated damage to the whole neurovascular system. Moreover, our work also highlights the complementary role of Mg^2+^ and CypD siRNA in protecting mitochondria and improving memory function in 5xFAD mice. These findings provide compelling evidence that halting the mitochondrial damage and restoring mitochondrial function will be a potential treatment for AD.

## Conflict of Interest

The authors declare no conflict of interest.

## Supporting information

Supporting Information

Supplemental Video 1

## Data Availability

The data that support the findings of this study are available in the supplementary material of this article.

## References

[1] M. Calvo‐Rodriguez , B. J. Bacskai , Trends Neurosci. 2021, 44, 136. 33160650 10.1016/j.tins.2020.10.004

[2] Alzheimers Dement. 2021, 17, 327.33756057

[3] W. Wang , F. Zhao , X. Ma , G. Perry , X. Zhu , Mol. Neurodegener. 2020, 15, 30. 32471464 10.1186/s13024-020-00376-6 PMC7257174

[4] T. Cali , D. Ottolini , M. Brini , Cell Calcium 2012, 52, 73. 22608276 10.1016/j.ceca.2012.04.015 PMC3396847

[5] H. Atamna , W. N. Frey , Mitochondrion 2007, 7, 297. 17625988 10.1016/j.mito.2007.06.001

[6] H. Xie , J. Guan , L. A. Borrelli , J. Xu , A. Serrano‐Pozo , B. J. Bacskai , J. Neurosci. 2013, 33, 17042. 24155308 10.1523/JNEUROSCI.1836-13.2013 PMC3807029

[7] R. X. Santos , S. C. Correia , X. Wang , G. Perry , M. A. Smith , P. I. Moreira , X. Zhu , J. Alzheimers Dis. 2010, 20, S401. 20463393 10.3233/JAD-2010-100666 PMC2923835

[8] K. J. Kopeikina , G. A. Carlson , R. Pitstick , A. E. Ludvigson , A. Peters , J. I. Luebke , R. M. Koffie , M. P. Frosch , B. T. Hyman , T. L. Spires‐Jones , Am. J. Pathol. 2011, 179, 2071. 21854751 10.1016/j.ajpath.2011.07.004 PMC3181340

[9] H. M. Wilkins , S. M. Carl , S. G. Weber , S. A. Ramanujan , B. W. Festoff , D. A. Linseman , R. H. Swerdlow , J. Alzheimers Dis. 2015, 45, 305. 25537010 10.3233/JAD-142334 PMC4605548

[10] M. Calvo‐Rodriguez , S. S. Hou , A. C. Snyder , E. K. Kharitonova , A. N. Russ , S. Das , Z. Fan , A. Muzikansky , M. Garcia‐Alloza , A. Serrano‐Pozo , E. Hudry , B. J. Bacskai , Nat. Commun. 2020, 11, 2146. 32358564 10.1038/s41467-020-16074-2 PMC7195480

[11] R. Parodi‐Rullan , J. Y. Sone , S. Fossati , J. Alzheimers Dis. 2019, 72, 1019. 31306129 10.3233/JAD-190357 PMC6917858

[12] Z. Huang , Q. Yan , Y. Wang , Q. Zou , J. Li , Z. Liu , Z. Cai , J. Alzheimers Dis. 2020, 78, 505. 33044180 10.3233/JAD-200519

[13] S. C. Cunnane , E. Trushina , C. Morland , A. Prigione , G. Casadesus , Z. B. Andrews , M. F. Beal , L. H. Bergersen , R. D. Brinton , S. de la Monte , A. Eckert , J. Harvey , R. Jeggo , J. H. Jhamandas , O. Kann , C. M. la Cour , W. F. Martin , G. Mithieux , P. I. Moreira , M. P. Murphy , K. A. Nave , T. Nuriel , S. Oliet , F. Saudou , M. P. Mattson , R. H. Swerdlow , M. J. Millan , Nat. Rev. Drug Discovery 2020, 19, 609. 32709961 10.1038/s41573-020-0072-x PMC7948516

[14] C. G. Monzio , A. Di Fonzo , S. Corti , G. P. Comi , N. Bresolin , E. Masliah , Mol. Neurobiol. 2020, 57, 2959. 32445085 10.1007/s12035-020-01926-1 PMC9047992

[15] Y. Han , X. Chu , L. Cui , S. Fu , C. Gao , Y. Li , B. Sun , Drug Delivery 2020, 27, 502. 32228100 10.1080/10717544.2020.1745328 PMC7170363

[16] S. T. N. Dos , S. S. Da , R. Arruda , K. S. Ugioni , P. B. Canteiro , S. G. de Bem , C. Mendes , P. Silveira , A. P. Muller , Mol. Neurobiol. 2020, 57, 926. 31612296 10.1007/s12035-019-01780-w

[17] J. M. Dowding , W. Song , K. Bossy , A. Karakoti , A. Kumar , A. Kim , B. Bossy , S. Seal , M. H. Ellisman , G. Perkins , W. T. Self , E. Bossy‐Wetzel , Cell Death Differ. 2014, 21, 1622. 24902900 10.1038/cdd.2014.72 PMC4158687

[18] I. G. Onyango , J. Dennis , S. M. Khan , Aging Dis. 2016, 7, 201. 27114851 10.14336/AD.2015.1007 PMC4809610

[19] J. Xu , J. G. Shamul , E. A. Kwizera , X. He , Nanomaterials 2022, 12, 743. 35269231 10.3390/nano12050743 PMC8911864

[20] M. P. Murphy , R. C. Hartley , Nat. Rev. Drug Discovery 2018, 17, 865. 30393373 10.1038/nrd.2018.174

[21] Du H , L. Guo , F. Fang , D. Chen , A. A. Sosunov , G. M. McKhann , Y. Yan , C. Wang , H. Zhang , J. D. Molkentin , F. J. Gunn‐Moore , J. P. Vonsattel , O. Arancio , J. X. Chen , S. D. Yan , Nat. Med. 2008, 14, 1097. 18806802 10.1038/nm.1868 PMC2789841

[22] I. Pilchova , K. Klacanova , Z. Tatarkova , P. Kaplan , P. Racay , Oxid. Med. Cell Longevity 2017, 2017, 6797460. 10.1155/2017/6797460 PMC5516748 28757913

[23] D. Gu , F. Liu , M. Meng , L. Zhang , M. L. Gordon , Y. Wang , L. Cai , N. Zhang , Ann. Clin. Transl. Neurol. 2020, 7, 1681. 32790155 10.1002/acn3.51155 PMC7480907

[24] J. H. de Baaij , J. G. Hoenderop , R. J. Bindels , Physiol. Rev. 2015, 95, 1. 25540137 10.1152/physrev.00012.2014

[25] S. Long , A. M. Romani , Austin J. Nutr. Food Sci. 2014, 2, 1051. 25839058 PMC4379450

[26] J. Tang , L. Li , C. B. Howard , S. M. Mahler , L. Huang , Z. P. Xu , J. Mater. Chem. B 2015, 3, 6805. 27213045 10.1039/C5TB00912J PMC4869335

[27] J. Li , Y. Yang , L. Huang , J. Controlled Release 2012, 158, 108. 10.1016/j.jconrel.2011.10.020 PMC3288896 22056915

[28] R. D. Bell , E. A. Winkler , I. Singh , A. P. Sagare , R. Deane , Z. Wu , D. M. Holtzman , C. Betsholtz , A. Armulik , J. Sallstrom , B. C. Berk , B. V. Zlokovic , Nature 2012, 485, 512. 22622580 10.1038/nature11087 PMC4047116

[29] B. Vafadari , A. Salamian , L. Kaczmarek , J. Neurochem. 2016, 139, 91. 10.1111/jnc.13415 26525923

[30] T. Ashleigh , R. H. Swerdlow , M. F. Beal , Alzheimers Dement. 2023, 19, 333. 35522844 10.1002/alz.12683

[31] J. W. Lustbader , M. Cirilli , C. Lin , H. W. Xu , K. Takuma , N. Wang , C. Caspersen , X. Chen , S. Pollak , M. Chaney , F. Trinchese , S. Liu , F. Gunn‐Moore , L. F. Lue , D. G. Walker , P. Kuppusamy , Z. L. Zewier , O. Arancio , D. Stern , S. S. Yan , H. Wu , Science 2004, 304, 448. 15087549 10.1126/science.1091230

[32] J. L. Huang , G. Jiang , Q. X. Song , X. Gu , M. Hu , X. L. Wang , H. H. Song , L. P. Chen , Y. Y. Lin , D. Jiang , J. Chen , J. F. Feng , Y. M. Qiu , J. Y. Jiang , X. G. Jiang , H. Z. Chen , X. L. Gao , Nat. Commun. 2017, 8, 15144. 28489075 10.1038/ncomms15144 PMC5436231

[33] T. Klecker , B. Westermann , Nat. Cell Biol. 2022, 24, 410. 35418623 10.1038/s41556-022-00889-w

[34] P. S. Minhas , A. Latif‐Hernandez , M. R. McReynolds , A. S. Durairaj , Q. Wang , A. Rubin , A. U. Joshi , J. Q. He , E. Gauba , L. Liu , C. Wang , M. Linde , Y. Sugiura , P. K. Moon , R. Majeti , M. Suematsu , D. Mochly‐Rosen , I. L. Weissman , F. M. Longo , J. D. Rabinowitz , K. I. Andreasson , Nature 2021, 590, 122. 33473210 10.1038/s41586-020-03160-0 PMC8274816

[35] J. L. Gollihue , C. M. Norris , Ageing Res. Rev. 2020, 59, 101039. 32105849 10.1016/j.arr.2020.101039 PMC7422487

[36] P. Racay , Cell Biol. Int. 2008, 32, 136. 17933560 10.1016/j.cellbi.2007.08.024

[37] H. Yang , C. Sibilla , R. Liu , J. Yun , B. A. Hay , C. Blackstone , D. C. Chan , R. J. Harvey , M. Guo , Nat. Commun. 2022, 13, 1582. 35332133 10.1038/s41467-022-29071-4 PMC8948191

[38] R. Quintana‐Cabrera , I. Manjarres‐Raza , C. Vicente‐Gutierrez , M. Corrado , J. P. Bolanos , L. Scorrano , Redox Biol. 2021, 41, 101944. 33780775 10.1016/j.redox.2021.101944 PMC8039725

[39] A. Rossi , P. Pizzo , R. Filadi , Biochim. Biophys. Acta, Mol. Cell Res. 2019, 1866, 1068. 30982525 10.1016/j.bbamcr.2018.10.016

[40] D. A. Nation , M. D. Sweeney , A. Montagne , A. P. Sagare , L. M. D'Orazio , M. Pachicano , F. Sepehrband , A. R. Nelson , D. P. Buennagel , M. G. Harrington , T. Benzinger , A. M. Fagan , J. M. Ringman , L. S. Schneider , J. C. Morris , H. C. Chui , M. Law , A. W. Toga , B. V. Zlokovic , Nat. Med. 2019, 25, 270. 30643288 10.1038/s41591-018-0297-y PMC6367058

[41] T. Zehnder , F. Petrelli , J. Romanos , F. E. De Oliveira , T. J. Lewis , N. Deglon , F. Polleux , M. Santello , P. Bezzi , Cell Rep. 2021, 35, 108952. 33852851 10.1016/j.celrep.2021.108952

[42] S. Han , M. Zhang , Y. Y. Jeong , D. J. Margolis , Q. Cai , Autophagy 2021, 17, 4182. 33757395 10.1080/15548627.2021.1907167 PMC8726713

[43] V. V. Gusel'Nikova , D. E. Korzhevskiy , Acta Naturae 2015, 7, 42. 26085943 PMC4463411

[44] D. H. Toffa , M. A. Magnerou , A. Kassab , D. F. Hassane , A. D. Sow , Neurochem. Int. 2019, 126, 195. 30905744 10.1016/j.neuint.2019.03.014

[45] J. Yu , M. Sun , Z. Chen , J. Lu , Y. Liu , L. Zhou , X. Xu , D. Fan , D. Chui , J. Alzheimers Dis. 2010, 20, 1091. 20413885 10.3233/JAD-2010-091444

[46] V. Sorrentino , M. Romani , L. Mouchiroud , J. S. Beck , H. Zhang , D. D'Amico , N. Moullan , F. Potenza , A. W. Schmid , S. Rietsch , S. E. Counts , J. Auwerx , Nature 2017, 552, 187. 29211722 10.1038/nature25143 PMC5730497

[47] A. R. Nelson , M. D. Sweeney , A. P. Sagare , B. V. Zlokovic , Biochim. Biophys. Acta 2016, 1862, 887. 26705676 10.1016/j.bbadis.2015.12.016 PMC4821735

[48] E. F. Fang , Y. Hou , K. Palikaras , B. A. Adriaanse , J. S. Kerr , B. Yang , S. Lautrup , M. M. Hasan‐Olive , D. Caponio , X. Dan , P. Rocktaschel , D. L. Croteau , M. Akbari , N. H. Greig , T. Fladby , H. Nilsen , M. Z. Cader , M. P. Mattson , N. Tavernarakis , V. A. Bohr , Nat. Neurosci. 2019, 22, 401. 30742114 10.1038/s41593-018-0332-9 PMC6693625

