# Supplementary material for: Nano‐Brake Halts Mitochondrial Dysfunction Cascade to Alleviate Neuropathology and Rescue Alzheimer's Cognitive Deficits
Source: Adv Sci (Weinh). 2023 Jan 26;10(7):2204596. doi: 10.1002/advs.202204596 (PMC9982524; doi:10.1002/advs.202204596)
Supplement: Supplementary file 1 — Supporting Information [file ADVS-10-2204596-s002.pdf]

Supporting Information

Nano-brake Halts Mitochondrial Dysfunction Cascade to Alleviate Neuropathology and Rescue Alzheimer's Cognitive Deficits

Qian Zhang[1], Qingxiang Song[1], Renhe Yu[1], Antian Wang, Gan Jiang, Yukun Huang, Jun Chen, Jianrong Xu, Dayuan Wang, Hongzhuan Chen, Xiaoling Gao**

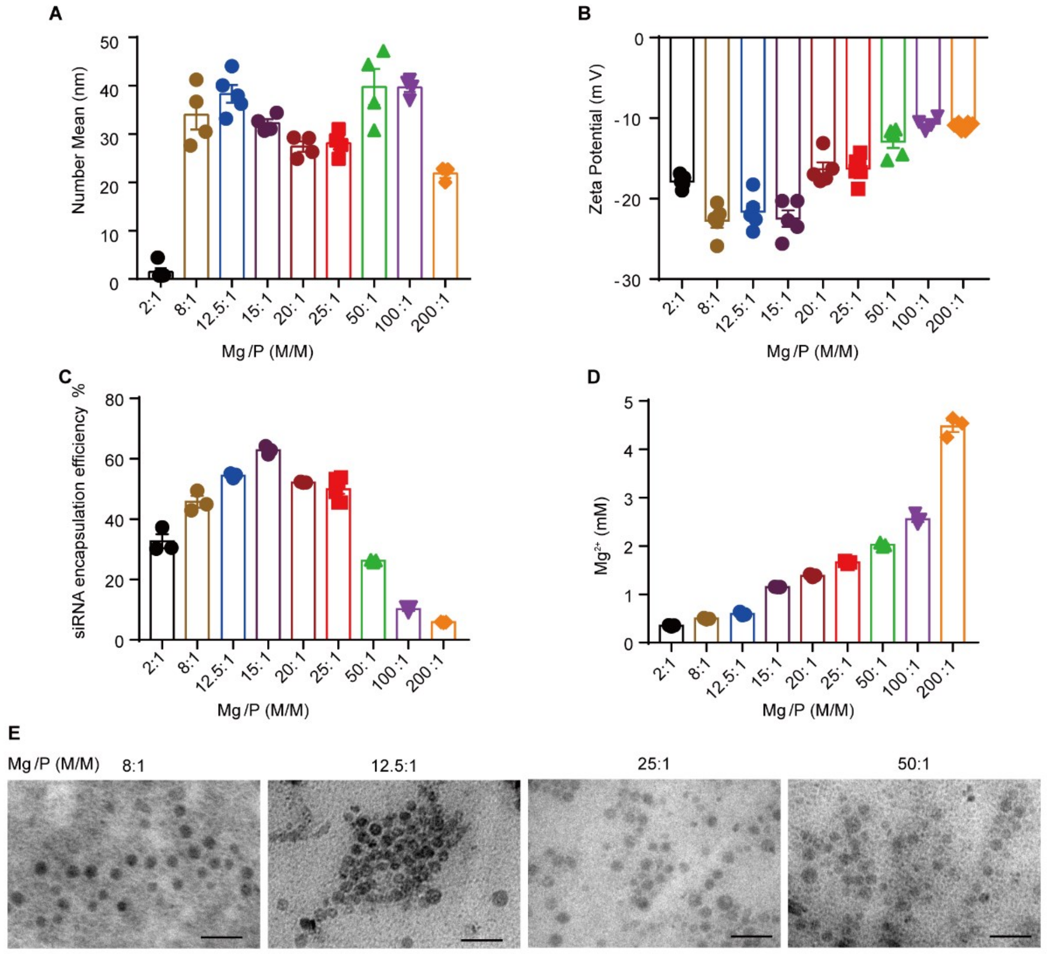

Figure S1. Optimizing the molar ratio of Mg^{2+} to $H_xPO_4^{x-3}$ (Mg/P) in Mg-siRNA core formulation. Number mean (A) and zeta potential (B) of Mg-siRNA core contained different Mg/P. Encapsulation efficiency of siRNA (C) and concentration of Mg^{2+} (D) loaded in the formulation at different magnesium and phosphate ions ratios. n=3. A-D, Data represent the mean ± SEM. E. Transmission electron microscope image of Mg-

siRNA core with different magnesium ion and phosphate ion ratios. Scale bar: 50 nm.

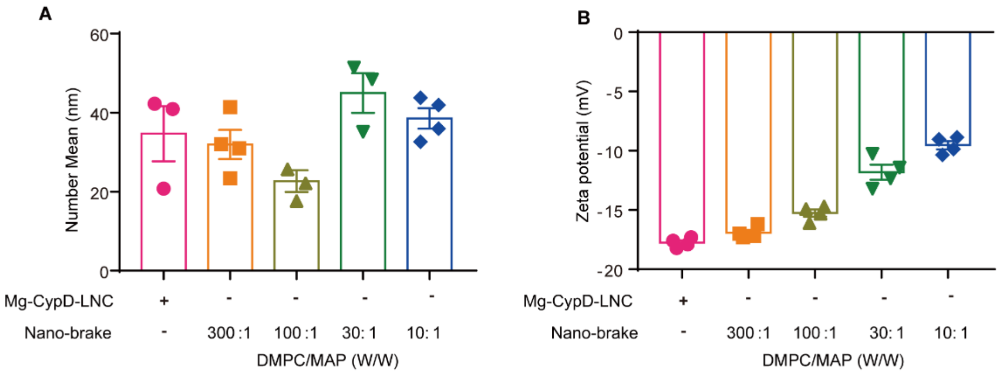

Figure S2. Optimization of the MAP-incorporated formulation. Number mean (A) and zeta potential (B) of Nano-brake contained different amounts of MAP (weight ratio). n=3-4. Data represent the mean ± SEM.

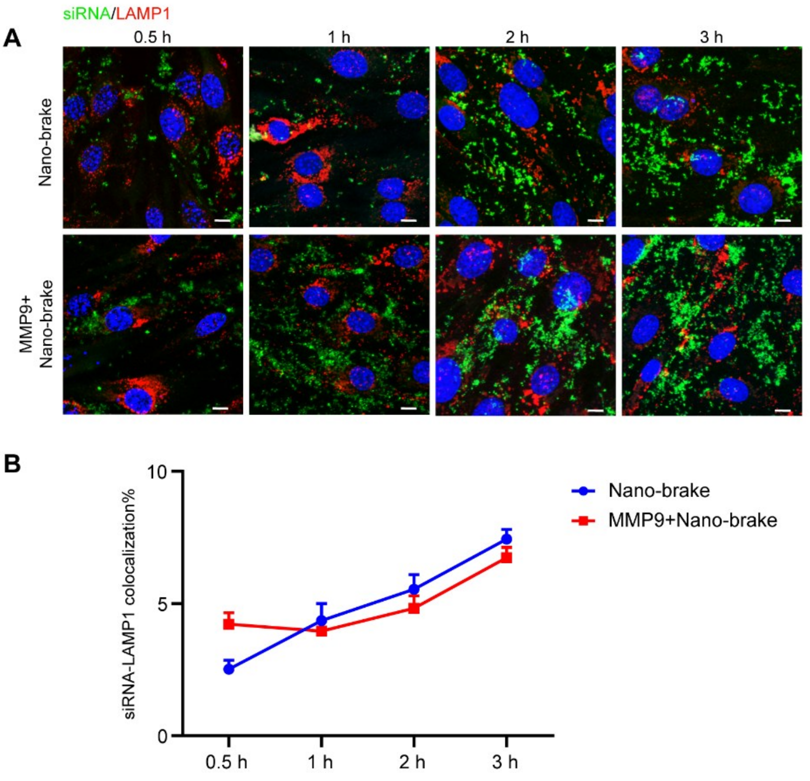

Figure S3. Nano-brake was not co-localized with lysosome in bEnd.3 cells. (A). Confocal images of bEnd.3 cells treated with Nano-brake or Nano-brake (pre-incubated

with MMP9 protein) for 0.5 h, 1 h, 2 h, and 3 h, respectively. siRNA (green), lysosome (LAMP1 characterization, red). Scale bar, 25 μm. (B). The co-localization index of lysosome and siRNA, n=4. Data represent mean ± SEM.

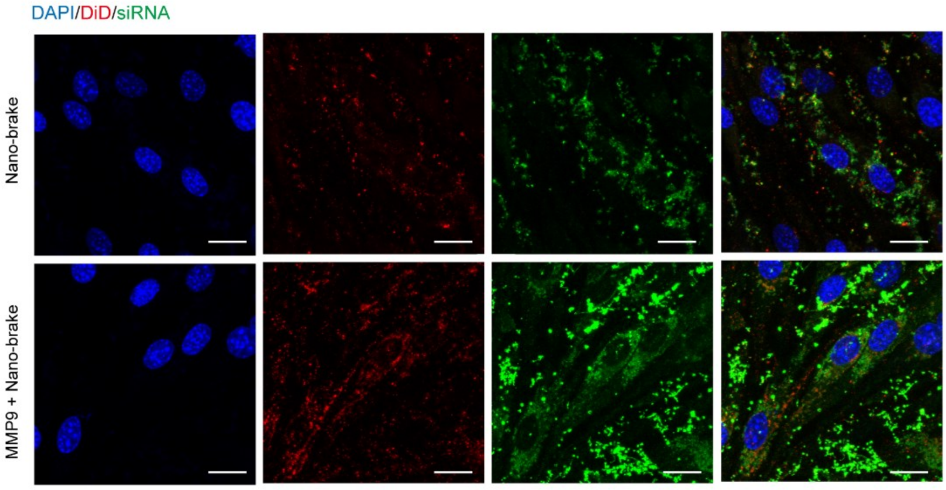

Figure S4. The distribution of Nano-brake in bEnd.3 cells. The bEnd.3 cells were incubated with FAM-CypD siRNA (green)-loaded DiD-Nano-brake (DiD labeled the carrier, red) with or without MMP9 pretreatment for 3 h. Scale bar, 20 μm.

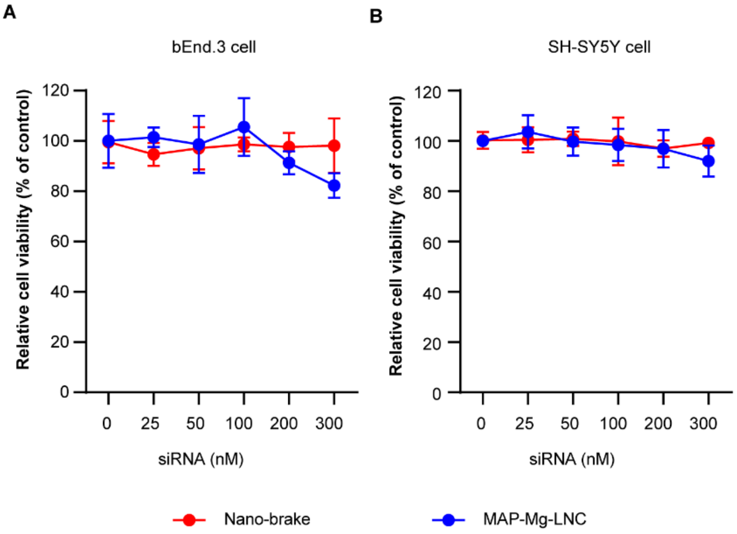

Figure S5. The toxicity of Nano-brake in bEnd.3 and SH-SY5Y cells were assessed by CCK8 assay. bEnd.3 (A) or SH-SY5Y (B) cells co-incubated with Nano-brake or MAP-Mg-LNC (NC-siRNA instead of CypD siRNA at 0, 25, 50, 100, 200, 300 nM (dose in

siRNA concentration) to test the toxicity of this carrier) for 24 h, respectively. Then, the cell viability was measured by CCK8 assay, n=3-4, Data represent the mean ± SD.

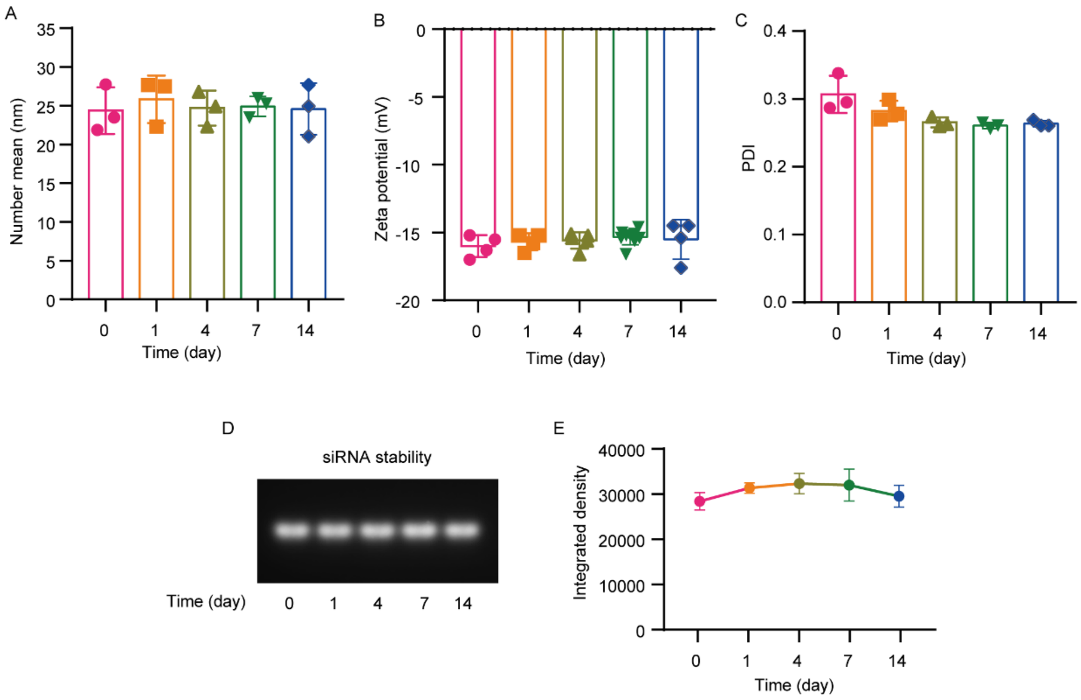

Figure S6. Nano-brake showed good storage stability. Number mean (A), zeta potential (B), and PDI (C) of Nano-brake after stored at 4°C for 0, 1, 4, 7, 14 days. (D), (E), Nano-brake protects siRNA from degradation. After stored at 4°C for 0, 1, 4, 7, 14 days, the remaining siRNA content in Nano-brake was determined. Data represent the mean ± SD. n=3.

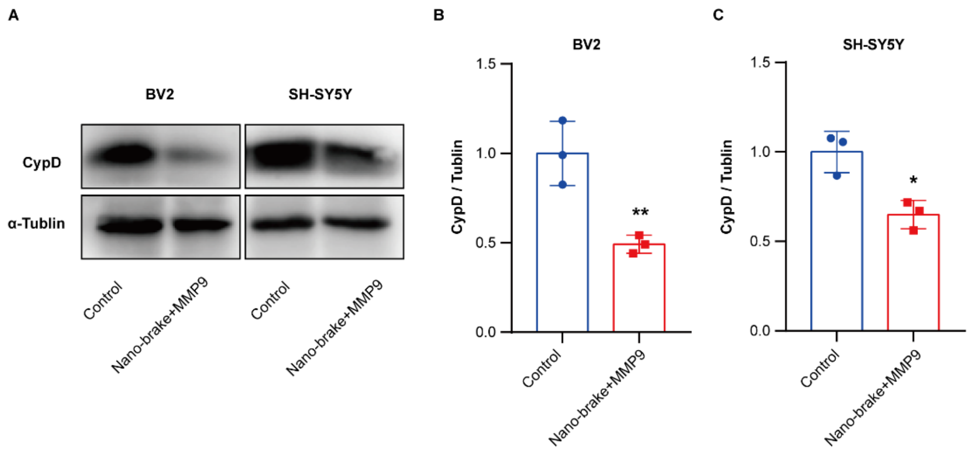

Figure S7. Nano-brake decreased CypD expression in BV2 and SHSY5Y cells. BV2 (A) or SH-SY5Y (B) cells co-incubated with Nano-brake (pretreated with MMP9) for 48 h at 100 nM (dose in siRNA concentration), then, the CypD expression was measured by Western Blot, n=3, Data represent the mean ± SD, *p < 0.05, **p < 0.01, significantly different with that of the DMEM control group.

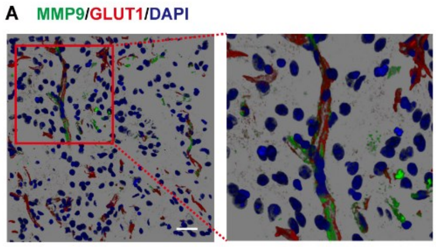

Figure S8. High expression of MMP9 in blood vessels of AD model mice. The expression and distribution of MMP9 in the cerebral vessels of 6-month-old 5xFAD mice**.** Scale bar, 50 μm.

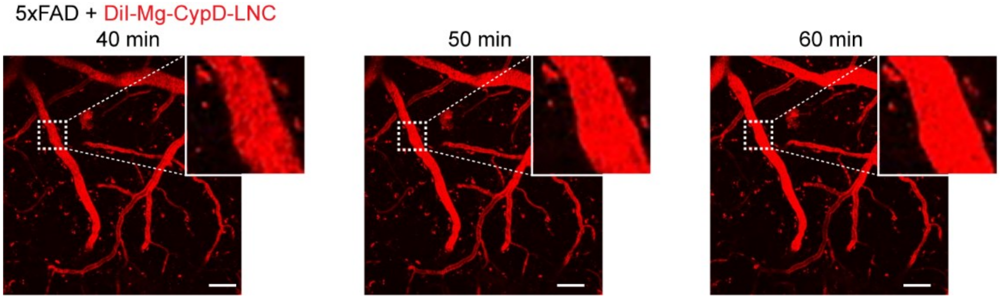

Figure S9. DiI-Mg-CypD-LNC did not target the damaged cerebral microvasculature or achieve intracerebral delivery in AD model mice. After administration of DiI labeled

Mg-CypD-LNC (41.3 μg/kg siRNA) via the tail vein of 6-month-old 5xFAD mice, the distribution of the DiI-Mg-CypD-LNC in the brain. Two-photon imaging images of DiI-Mg-CypD-LNC distributed in the cerebral vessels of 5xFAD mice. Scale bar, 40 μm.

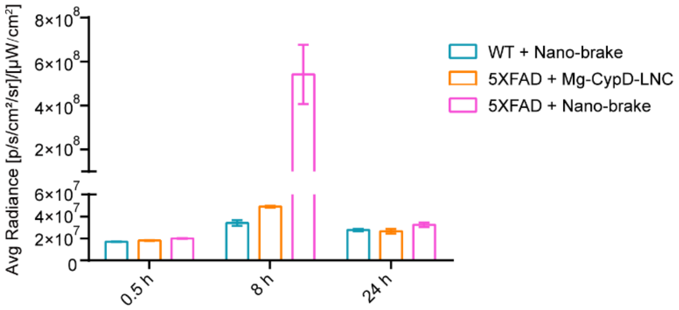

Figure S10. The brain entry efficiency of DiI-Nano-brake increased in 5xFAD mice. Six month-old 5xFAD mice and littermate WT mice were injected with DiR fluorescent-labeled Nano-brake and Mg-CypD-LNC (82.5 μg/kg siRNA) via the tail vein, respectively. n=3-4. The statistical assay of DiR fluorescent intensity of the brain in Fig. 2E was analyzed by IVIS Spectrum/CT imaging system. Data represent the mean ± SEM.

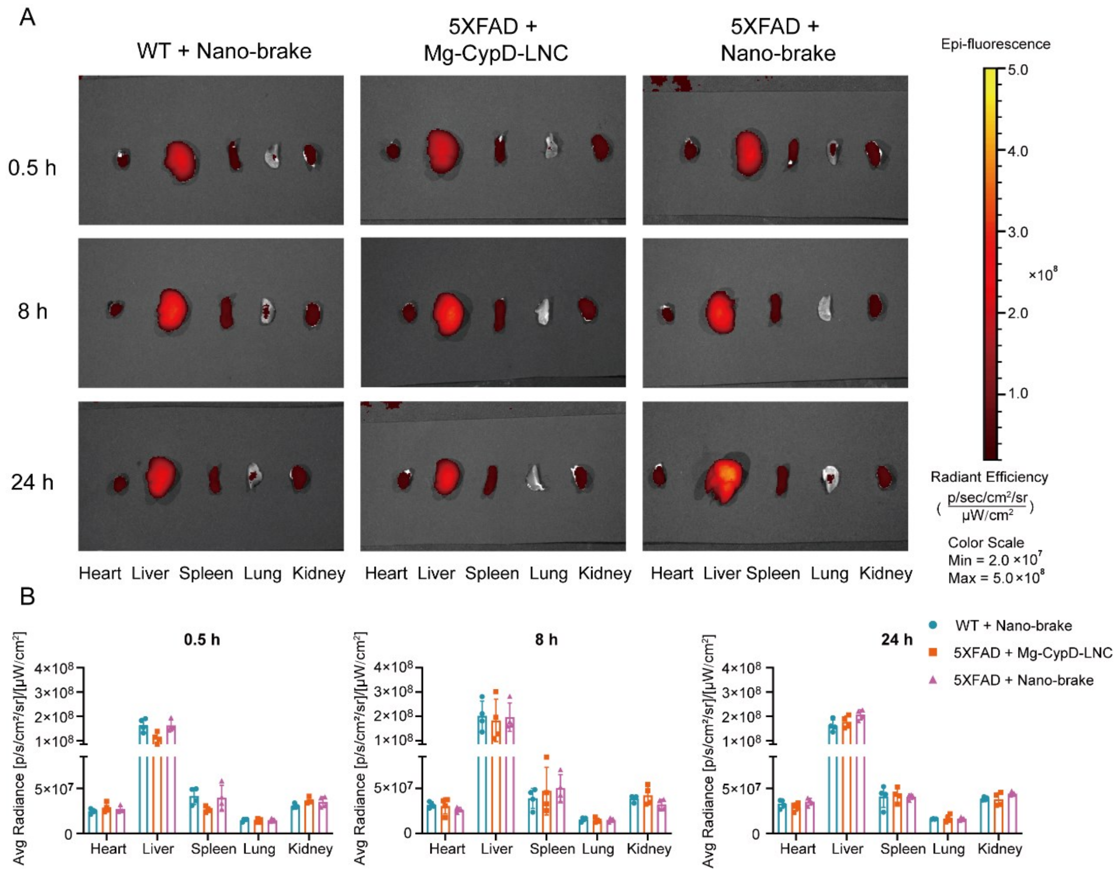

Figure S11. DiR-Nano-brake showed similar distribution profiles in the peripheral organs of 5xFAD mice with Mg-CypD-LNC. Six-month-old 5xFAD mice and littermate WT mice were injected with DiR-labeled Nano-brake and Mg-CypD-LNC (82.5 μg/kg siRNA) via the tail vein, respectively. A. The fluorescent images of the organs were taken via an IVIS Spectrum/CT imaging system. B. Quantification of DiR fluorescence intensity in heart, liver, spleen, lung, and kidney of 5xFAD mice. n=4.

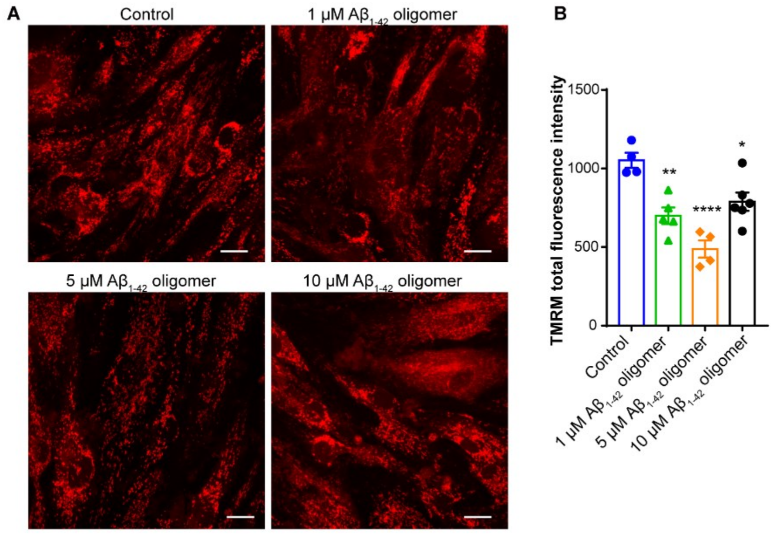

Figure S12. A$\beta_{1\text{-}42}$ oligomers reduce mitochondrial membrane potential. bEnd.3 cells were incubated with A$\beta_{1\text{-}42}$ oligomers at various concentrations for 48 h, and TMRM was used to characterize mitochondria membrane potential. A. The laser confocal images of mitochondria characterized by TMRM in different treatment groups, scale bar, 20 μm. B. The statistic chart of TMRM fluorescence intensity in various concentrations A$\beta_{1\text{-}42}$ oligomers treatment groups. Data represent the mean ± SEM, n=4-5. *$p < 0.05$, **$p < 0.01$, ****$p < 0.0001$, significantly different with that of the DMEM solvent control.

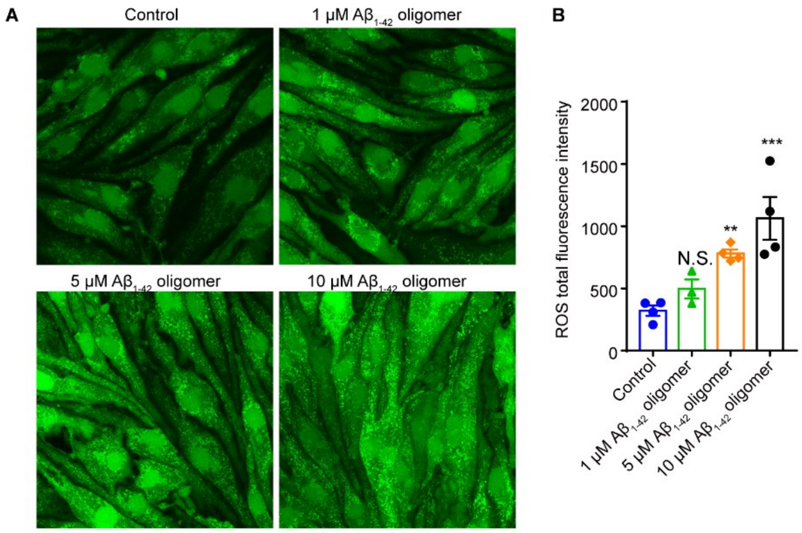

Figure S13. A$\beta_{1\text{-}42}$ oligomer increased intracellular ROS level. After being incubated with Aβ oligomer at various concentrations for 48 h, the ROS in bEnd.3 cells were

determined by fluorescent dye (DCFH-DA; green). A. Laser confocal images of ROS expression in different concentrations Aβ_{1-42} oligomers treatment groups, scale bar, 20 µm. B. ROS fluorescence probe intensity in different treatment groups. Data represent the mean ± SEM, n=3-4. **p < 0.01, ***p < 0.001, significantly different with that of the DMEM solvent control. N.S., not significant compared to the DMEM solvent control.

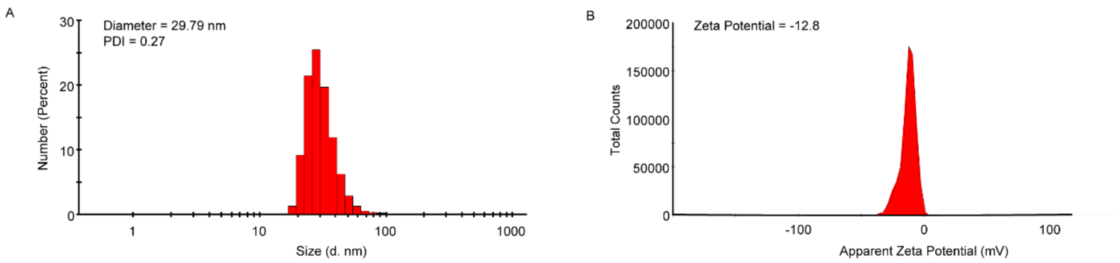

Figure S14. The size distribution (A) and zeta potential (B) of MAP-Ca-CypD-LNC as detected by dynamic light scattering (DLS).

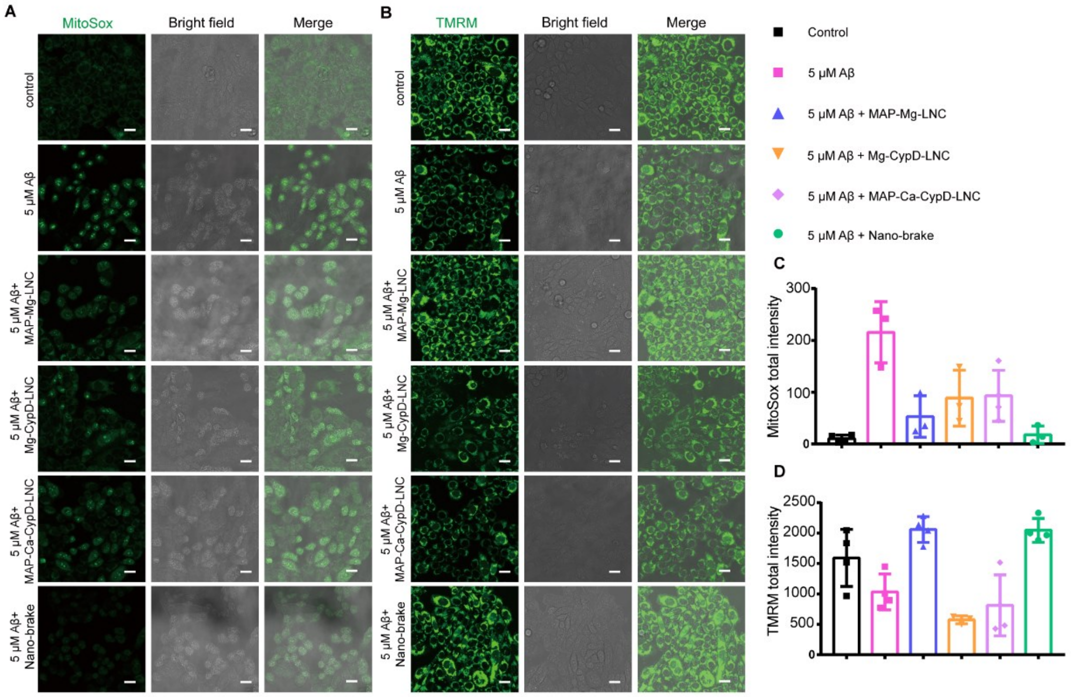

Figure S15. SH-SY5Y cells were co-incubated with Aβ_{1-42} oligomer (5 µM) and different formulations (Mg-CypD-LNC, MAP-Mg-LNC, MAP-Ca-CypD-LNC, Nano-brake) for 48 h, respectively. A-D, Nano-brake alleviates Aβ_{1-42}-induced mitochondrial

membrane potential decrease and superoxide production. A. The confocal images of MitoSox characterized mitochondria ROS. Scale bar, 20 μm. B. The laser confocal images of mitochondrial membrane potential were characterized by TMRM, scale bar, 20 μm. C. The statistical diagram of superoxide fluorescence intensity. D. The fluorescence intensity of TMRM. Data represent the mean ± SD, n=3-5.

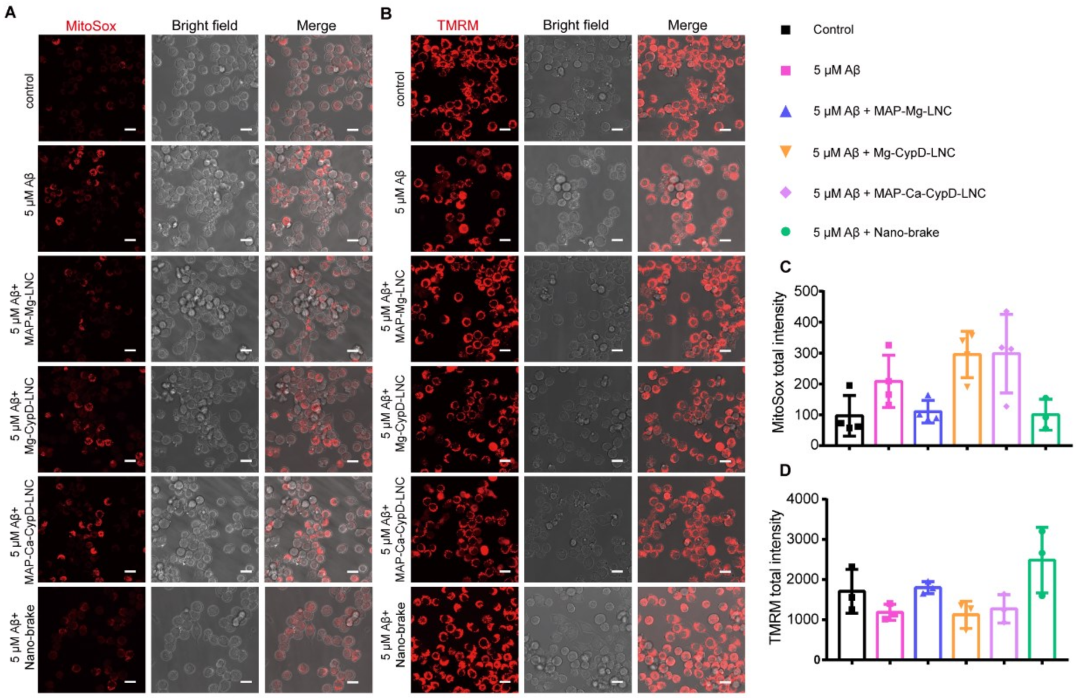

Figure S16. BV2 cells were co-incubated with $A\beta_{1-42}$ oligomer (5 μM) and different formulations (Mg-CypD-LNC, MAP-Mg-LNC, MAP-Ca-CypD-LNC, Nano-brake) for 48 h, respectively. A-D, Nano-brake alleviates $A\beta_{1-42}$-induced mitochondrial membrane potential decrease and superoxide production. A. The confocal images of MitoSox characterized mitochondria ROS. Scale bar, 20 μm. B. The confocal images of mitochondrial membrane potential were characterized by TMRM, scale bar, 20 μm. C. The fluorescence intensity of superoxide. D. The statistical diagram of TMRM fluorescence intensity. Data represent the mean ± SD, n=3-5.

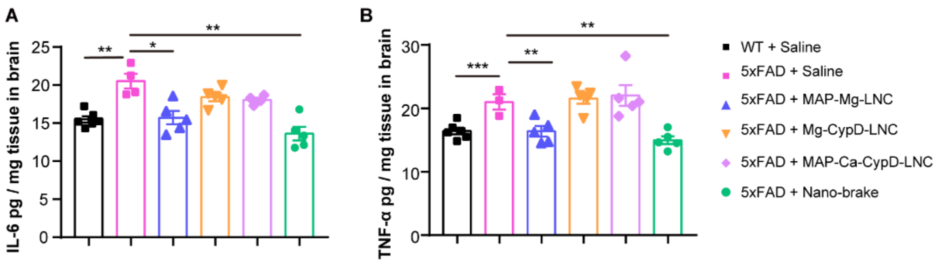

Figure S17. Nano-brake reduces neuroinflammation. Six-month-old 5xFAD mice were treated with Nano-brake, MAP-Mg-LNC, Mg-CypD-LNC and MAP-Ca-CypD-LNC (82.5 μg/kg siRNA) for 4 weeks. WT mice and 5xFAD mice treated with saline were set as normal and negative controls, respectively. A. The contents of IL-6 in the cortex of mice in different treatment groups. B. The contents of TNF-α in the cortex of mice in various treatment groups. Data represent the mean ± SD, n=3-5. *p < 0.05, **p < 0.01, ***p < 0.001, significantly different with that of saline treatment group of 5xFAD mice.

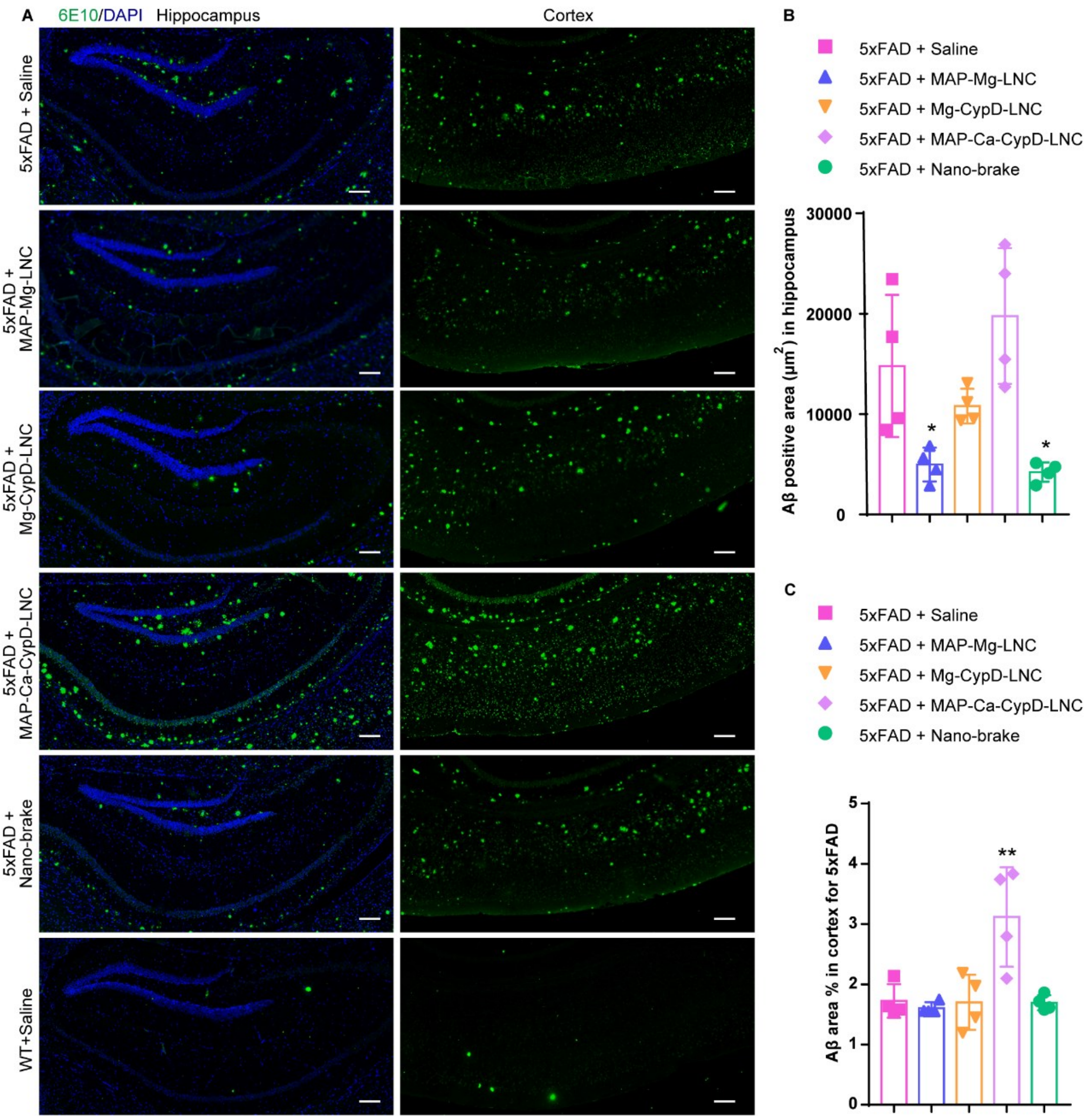

Figure S18. Nano-brake reduced Aβ deposition in the brain of 5xFAD mice. Six-month-old 5xFAD mice were treated with Nano-brake, MAP-Mg-LNC, Mg-CypD-LNC and MAP-Ca-CypD-LNC (82.5 μg/kg siRNA) for 4 weeks, WT mice and 5xFAD mice treated with saline served as the normal and negative controls, respectively. A. The immunohistochemical images of Aβ in the hippocampus of mice in different treatment groups, scale bar, 100 μm. B. Statistics of Aβ positive area in the hippocampus of mice in different treatment groups. C, Statistics of Aβ area percent in the cortex for 5xFAD mice. Data represent the mean ± SEM, n=4. *$p < 0.05$, **$p < 0.01$, significantly different from the group of 5xFAD mice treated with saline.

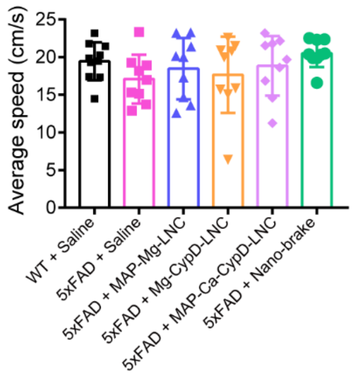

Figure S19. Nano-brake did not change the swimming speed of 5xFAD mice. Six-month-old 5xFAD mice were administered with Nano-brake, MAP-Mg-LNC, Mg-CypD-LNC and MAP-Ca-CypD-LNC (82.5 μg/kg siRNA) for 4 weeks. WT mice and 5xFAD mice treated with saline were set as normal and negative controls, respectively. There was no difference in the swimming speed of mice from each group during the MWM test, n=9.

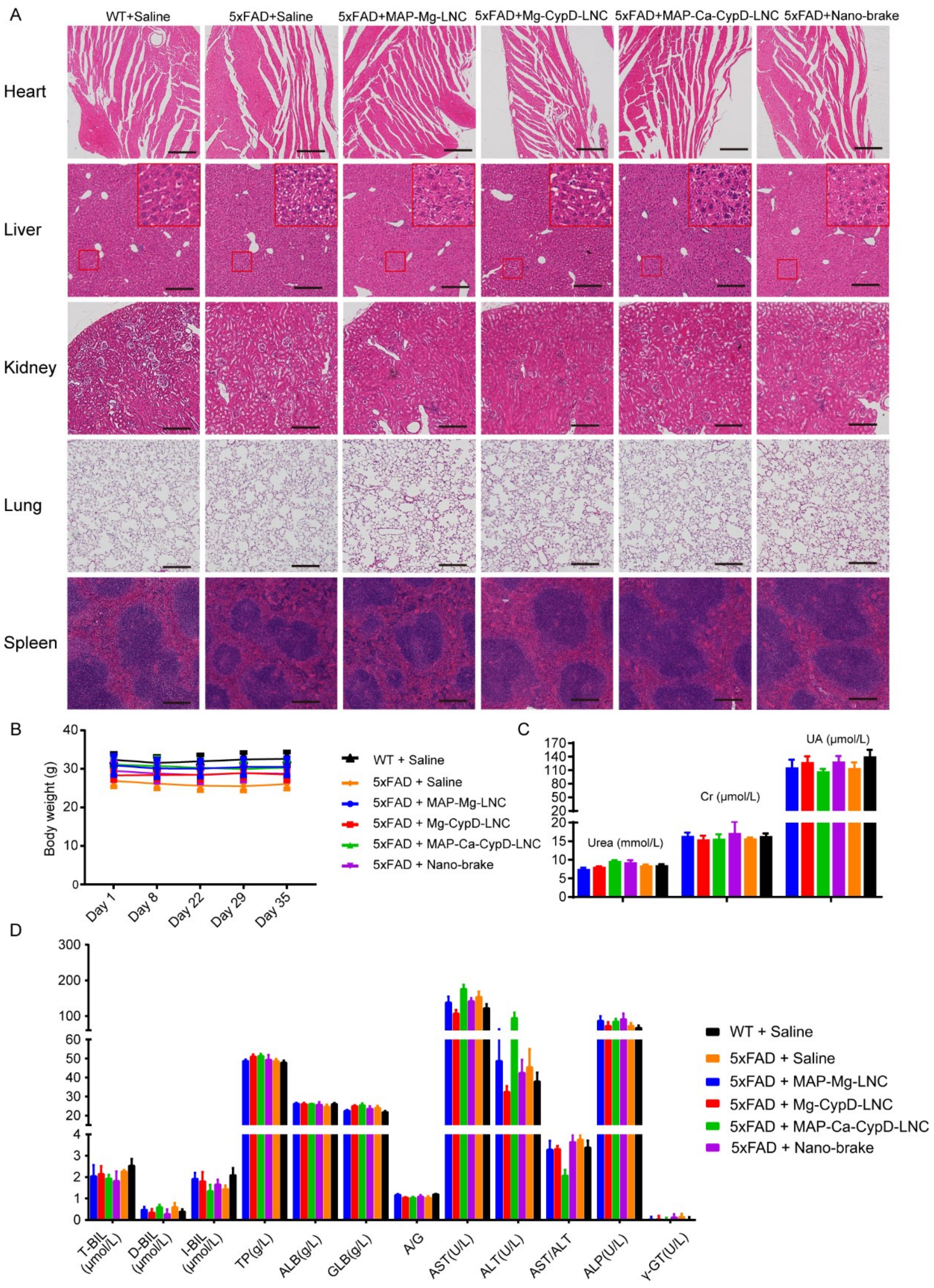

Figure S20. Biosafety evaluation of different treatments. Six-month-old 5xFAD mice were treated with Nano-brake, MAP-Mg-LNC, Mg-CypD-LNC and MAP-Ca-CypD-LNC (82.5 μg/kg siRNA) for 4 weeks. WT mice and 5xFAD mice treated with saline were set as normal and negative controls, respectively. A. H&E staining images of heart, liver, kidney, lung and spleen to access the safety of different nanoformulations

treatments. Scale bars, 200 μm. B. During the nanoformulations treatment and behavior test, the body weight of mice was monitored (n=9 mice). C. Quantification of renal injury marker blood urea nitrogen (Urea), blood serum creatinine (Cr), blood uric acid (UA) of different treatments, n=6-7. D. Quantification of liver injury marker total bilirubin (T-BIL), direct bilirubin (D-BIL), indirect bilirubin (I-BIL), total protein (TP), albumin (ALB), globulin (GLB), ALB/GLB (A/G), alkaline phosphatase (ALP), aminotransferase (ALT), aspartate aminotransferase (AST), AST/ALT and γ-glutamyl transpeptidase (γ-GT) of different treatments, n=6-7.

Video S1. After administration of DiI labeled Nano-brake (41.3 μg/kg siRNA) via the tail vein of 6-month-old 5xFAD mice. Two-photon imaging video of Nano-brake distributed on the cerebral vessels of 5xFAD mice from 40 min to 70 min. The white arrow showed higher adhesion along and permeation across the cerebral vessels. Scale bar, 40 μm.

4. Experimental Section

Animals. 5xFAD mice were provided by the Jackson Laboratory (Bar Harbor, ME USA) and bred in the specific pathogen-free animal facility (SPF). The animals were raised in the SPF animal facility with free access to water and food at 22 ± 2°C and a light-dark cycle of 12 hours. All animal experiments were approved by the Shanghai Jiao Tong University School of Medicine Animal Experimentation Ethics Committee (License, SYXK(沪)2018-0027) and Institutional Animal Care and Use Committee (IACUC) (Project license, A-2019-021).

Preparation of Nano-brake. Magnesium phosphate core was loaded with CypD siRNA (Mg-siRNA core) using a reverse water-in-oil microemulsion method, referring to our previous calcium phosphate core preparation method[1]. As shown in Figure 1A, 50 μL of 50 μM siRNA and 300 μL of 0.625 M $MgCl_2$ were dispersed in 10 mL Igepal CO-520 / cyclohexane (7/3 V/V) solution to form a well dispersed microemulsion. Three

hundred microliters of 12.5 mM Na_2HPO_4 and 50 µL of 50 µM siRNA were added to 10 mL Igepal CO-520 / cyclohexane solution to prepare the phosphate part. DOPA (20 mM) chloroform solution (100 µL) was dropped into the above phosphate phase. The above two Igepal CO-520 / cyclohexane solutions were mixed and stirred for 40 min. Then, 20 mL ethanol was added for emulsion breaking. The mixture was centrifuged at 12,000 g for 20 min, thus the surfactant of this mixture was removed carefully. After two thorough washes with ethanol, the pellets were dissolved in 2 mL chloroform and named Mg-siRNA core. To prepare CypD siRNA-loaded lipid nanocarrier, 750 µL the above Mg-siRNA core, 4 mg DMPC, and 2 mL chloroform were mixed and dried using a Büchi Rotavapor vacuum at 100 rpm for 1 h. The above lipid film was rehydrated with 4 mL saline. The liposome solution (Mg-CypD-LNC) was briefly sonicated to decrease its size. For the preparation of Nano-brake, MAP (Sequence, AC-FAEKFKEAVKDYFAKFWD-GSG-RRRRRRRRR-PVGLIG-EGGEGGEGG, GL BioChem Shanghai, China) was incubated with Mg-CypD-LNC (at DMPC: MAP=100: 1, weight ratio) for 12 h at 4°C. MAP-Mg-LNC and MAP-Ca-CypD-LNC were prepared with the same method only with NC siRNA or using calcium phosphate instead of magnesium phosphate. Similarly, DiI and DiR-labelled nanoformulations were prepared with the above procedure by adding DiI (0.5% for *in vitro* and 4% for *in vivo* use) or DiR (4%) to the DMPC solution.

Characterization of Nano-brake. The nanocarriers' particle size and zeta potential were evaluated using a Zeta-sizer Nano-ZS90 system. Nano-brake were negative stained with 1.5% sodium phosphotungstate solution and characterized by TEM (JEOL, JEM-1400, Japan).

To evaluate the siRNA encapsulation efficiency of Mg-siRNA-LNC, cy3-siRNA was used as the fluorescent indicator. For the analysis, Mg-cy3-siRNA-LNC was dissolved in pH 7.8 Tris lysis buffer containing 2 mM EDTA and 0.05% Trixton X-100. They were then incubated at 65°C for 10 min to release the entrapped siRNA before being measured by the fluorescence microplate reader.

The Mg^{2+} level in the Nano-brake was quantitatively measured by Magnesium Assay Kit (Nanjing Jiangcheng Bioengineering Institute, China) based on the manufacturer's protocols.

The stability of Nano-brake in serum. Naked CypD siRNA, Nano-brake, and Mg-CypD-LNC were incubated for 0, 0.5, 2, 4 and 8 h in 10% fetal bovine serum (FBS). They were then analyzed by electrophoresis using 2% agarose gel to monitor the remaining siRNA content.

The storage stability of Nano-brake. After stored at 4°C for 0, 1, 4, 7, 14 days, the size, zeta potential and PDI of Nano-brake were determined by Zeta-sizer Nano-ZS90 system. The siRNA in Nano-brake was analyzed by electrophoresis using agarose gel to test the remaining siRNA content.

Nano-brake was pretreated with MMP9 protein to cleave MAP. In order to activate MMP9 protein, a mixture of 10 μL 100 μg/mL MMP9 protein (Sino Biological, Beijing, China. Cat: 10327-HNAH), 1 μL 100 μM P-aminophenylmercury acetate (Sigma-Aldrich, Cat NO. A9563), and 1.1 μL 100 mM $CaCl_2$ was co-incubated for 12 h at 37°C. The above-mentioned activated MMP9 protein (containing 1 μg MMP9 protein) was co-incubated with 250 μL Nano-brake (containing 0.25 mg DMPC) for 2 h at 37°C to cleave MAP.

Cell culture and cellular uptake of DiI-Nano-brake. bEnd.3 cells (Shanghai Type Culture Collection of the Chinese Academy of Sciences, China.) were seeded in a 96-well plate in DMEM medium containing 10% FBS, 100 μg/mL streptomycin, 100 units/mL penicillin and 1% non-essential amino acids at the density of 5×10^3 cells/well in a 37°C, 5% CO_2 humidified incubator to 80% confluency. The cells were incubated with DiI-Nano-brake (at 5 μg/mL DMPC) in DMEM for 3 h at 37°C. After rinsing with PBS, the cells were fixed with a 4% formaldehyde solution. Following nuclei staining with Hoechst, the above cells were quantitatively analyzed via an HCS instrument (Thermo Scientific Cellomics, Thermo, USA) to evaluate the cellular uptake of DiI-Nano-brake according to the previous method[2].

The toxicity of Nano-Brake was assessed by CCK8 assay. The toxicity of Nano-brake in bEnd.3 and SH-SY5Y cells (purchased from the Type Culture Collection of the Chinese Academy of Sciences) was evaluated by using the CCK8 assay. Briefly, the cells were seeded in a 96-well plate at the density of 5,000 cells per well. After 24 h, Nano-brake or MAP-MgP-NC-siRNA-LNC were added into wells and co-culture for 24 h at the siRNA concentration 0, 25, 50, 100, 200, 300 nM, respectively. After removing the Nano-brake or MAP-Mg-LNC, 200 μL CCK8 (containing 20 μL CCK8 diluted with 180 μL DMEM) was added into each well and incubated for 1 h (for bEnd.3 cells), 0.5 h (for SH-SY5Y cells), respectively. Then, the plates were subjected to a microplate reader for cell viability assay at the wavelength, 450 nm.

Detection of d9-DMPC nano-brake in the brain. The deuterium isotope labeled d9-nano-brake, or d9-Mg-CypD-LNC in the brain of mice was quantified by LC-MS/MS according to our previous method[3, 4]. Briefly, d9-Nano-brake or d9-Mg-CypD-LNC was prepared using the same method by replacing 1 mg DMPC with 1 mg d9-DMPC during the LNC preparation process. Six months old 5xFAD and WT littermates mice were administered by caudal vein at a dose of 82.5 μg/kg siRNA and grouped as follows: A) 5xFAD + d9-Nano-brake; B) 5xFAD + d9-Mg-CypD-LNC; C) WT + d9-Nano-brake. The brain homogenates were extracted using methyl *tert butyl* ether, and 13:0 PC was chosen as the internal standard. The concentration of d9-DMPC was determined in positive ionization mode using a triple-quadrupole mass spectrometer (AB Sciex API-4000), equipped with an electrospray ionization source (ESI). Chromatography analysis was performed in a C18 column (5.0 μm, 4.6 mm × 100 mm, Angela Technology, Tianjin, China), and the mobile phase consist of a ratio of 85:15 (V/V) isopropanol and water (containing 0.1% formic acid and 5 mM ammonium formate) with 0.5 mL/min of flow rate. The ionization source parameters were set as turbo heater gas pressure at 60 psi, turbo heater temperature at 600°C, curtain gas pressure at 20 psi, nebulizer gas pressure at 60 psi, the ion-spray voltage at 4500 v, collision gas pressure at 4 psi. The optimized collision energy was 41 V, and optimized decluttering potential

was 170 V. The multiple reaction monitoring (MRM) mode was applied to detect the IS and target ions, 13:0 PC was m/z 650.5/184.1 and d9-DMPC was m/z 687.5/193.1, respectively.

The proportion of administered dose per gram (%ID/g) was used to express the remaining amount of Nano-brake in the brain:

$$\%ID/g = \frac{amount\ of\ Nano\!-\!brake\ in\ the\ brain}{(amount\ of\ Nano\!-\!brake\ administrated) * (mouse\ body\ weight)} * 100\%$$

Western blot. For cell, bEnd.3 cells were cultured in a 6-well plate at the density of 2.5 × 10^5 cells/well. Following culturing for 24 h, the cells were incubated with DMEM control, Mg-CypD-LNC (pre-incubating with MMP9 protein), Nano-brake and Nano-brake (pre-incubating with MMP9 protein), respectively, for 48 h at a concentration of 100 nM siRNA. And then, CypD expression was evaluated by western blot as described previously[5]. For western blot brain tissue sample preparation, the brain tissue and RIPA lysis solution (containing 1 mM PMSF) were added to the grinding tube with a weight-to-volume ratio of 1:3, and then grinding magnetic beads were added. After that, the tubes were placed in the tissue grinding homogenizer and fully cracked for 3 min to acquire the brain homogenate. The 20–40 μg of cell or brain tissue lysates were electrophoresed using SDS–polyacrylamide gels and then transferred to PVDF membranes. The membranes were blocked in skimmed milk (5%), incubated with primary antibodies at 4°C overnight, including anti-CypD, anti-TOM20, anti-Drp1 and anti-OPA1, and then detected with IgG fluorescent secondary antibody. Finally, the Odyssey Fc Dual-Mode Imaging System(LI-COR Biosciences) was used to visualize the membranes and Image J Fiji was used to analyze. The detection of CypD expression in SH-SY5Y cells and BV-2 cells (from the Type Culture Collection of the Chinese Academy of Sciences) are similar to bEnd.3 cells, and the CypD and tubulin primary antibodies were detected with IgG HRP secondary antibody.

Small animal tomographic optical imaging and in vivo multiphoton imaging of cerebral vessels. Drug treatment: 5xFAD and WT littermates mice, 6 months old, were treated by caudal vein with a dose of 82.5 μg/kg siRNA, and grouped as A) 5xFAD +

4%-DiR-Nano-brake; B) 5xFAD + 4%-DiR-Mg-siRNA-LNC; C) WT + 4%-DiR-Nano-brake. The mice were anesthetized after the administration for 0.5 h, 4 h, 8 h, and 24 h. A small animal tomographic optical imaging system (IVIS Spectrum/CT imaging system, PerkinElmer, USA) was applied to test the fluorescence intensity of nanocarriers in the brain of mice.

To visualize Nano-brake binding to cerebral vessels, *in vivo* multi-photon imaging was applied to observe cerebral vessels referring to our previous method[6]. Briefly, ketamine (100 mg/kg) and midazolam (5 mg/kg) were intraperitoneally administered to the mice to maintain general anesthesia. Then, a cranial window was opened in the parietal bone, and thin grass was carefully placed and fixed with dental cement. DiI-labeled Nano-brake and Mg-CypD-LNC (41.3 μg/kg siRNA) were administrated through the tail vein. The cerebral vessels were imaged using a two-photon imaging system (FVMPE-RS, Olympus, Japan) equipped with a water-immersion objective (25 ×, NA 1.05; Olympus, Japan) and a Mai Tai laser (Spectra-Physics) at the excitation wave of 900 nm.

Mitochondrial damage protection of bEnd.3. To detect ROS, at 37°C, bEnd.3 cells were incubated with 100 μM DCFH-DA for 20 min, and then, were washed 3 times with a DMEM culture medium to remove the extracellular DCFH-DA fully. A laser confocal microscope was applied to acquire images at 488 nm excitation wavelength and 525 nm emission wavelength settings.

To detect mitochondrial membrane potential detection, similar to ROS detection, bEnd.3 cells were incubated with 300 nM TMRM for 5 min at 37°C, and washed 3 times. Living cells were also observed and imaged by laser confocal microscope.

To detect mitochondria superoxide, the cells were incubated with 5 mM MitoSOX for 5 min at 37°C and then was washed and imaged.

Mitochondrial superoxide and mitochondrial membrane potential detection of these two cell lines are similar to bEnd.3 cells.

TEM analysis of bEnd.3 cell mitochondria. To examine the ultrastructure of

mitochondria, bEnd.3 cells were incubated with 5 μM Aβ_{1-42} oligomer and Mg-CypD-LNC, MAP-Mg-LNC, MAP-Ca-CypD-LNC and Nano-brake for 48 h, respectively. After that, the cells were washed twice with PBS, fixed in 2.5% glutaraldehyde, and then stained with OsO4 as described before[7]. Finally, the ultra-thin section was analyzed by a Hitachi 7600 electron microscope.

Enzyme-linked immunosorbent assay (ELISA). ELISA kits (Multisciences Biotech, Hangzhou, China) were used to determine the TNF-α and IL-6 levels of the cortex homogenate based on the manufacturer's instructions and our previous method. ATP Assay Kit was used to quantitatively measure the ATP levels (Beyotime, Shanghai, China).

Drug Treatment. Six-month-old male 5xFAD mice were randomized into 5 groups and daily intravenously administered with saline, Nano-brake, MAP-Mg-LNC, Mg-CypD-LNC and MAP-Ca-CypD-LNC (82.5 μg/kg siRNA) for 4 weeks, respectively. The saline-treated wild-type littermates of the same age were set as the normal control.

Immunofluorescence and immunohistochemical analysis. Immunofluorescence analyses were performed based on our previous method[2]. The mice were euthanized as above mentioned and were perfused with 0.1 M PB and 4% formaldehyde solution for fixing brain tissue. Afterward, the brain was further preserved in 4% formaldehyde solution for 36 h, embedded in paraffin, and sectioned at a thickness of 4 μm. The brain slices were then immersed in citric acid for 15 min, and incubated with 3% H_2O_2 to quench the endogenous tissue peroxidase. The slices were then incubated with primary antibody overnight at 4°C. Primary antibodies including rabbit anti-GLUT1 (1:200, Cat#ab115730, Abcam), rabbit anti-Iba-1 (1:100, Cat#ab178847, Abcam), rabbit anti-GFAP (1:200, Cat#ab7260, Abcam) and rabbit anti-NeuN (1:200, Cat#ab177487, Abcam), were used to stain the cerebral vascular endothelia, microglia, astrocytes and neuron, respectively. The antigens were detected by fluorescent-labeled secondary antibodies, including donkey anti-rabbit Alexa Fluor 568 (Cat#A10042, Life tech) (1:2000), goat-anti-mouse Alexa Fluor 488 (Cat#A-10680, Life tech) (1:2000), and

observed under a laser confocal microscope. For immunohistochemical analysis, secondary antibodies Supervision™ Universal (Anti-Mouse/Rabbit) Detection Reagent (HRP) were used.

Cognitive function assessment. Morris Water Maze (MWM) test. The MWM test was carried out based on the previously described[2]. The training procedure lasted for four days, and the mice were trained 4 times each day. If the mice got the platform within 90 s, they were left to stay there for 10 s. Otherwise, they were led to the platform and remained for 30 s to bear in mind the platform location. On the probe trial day, let the mice swim in the pool without the platform. All analysis was recorded and analyzed by a tracking system (Shanghai Jiliang Software Technology, China).

Novel object recognition (NOR) test. The NOR test was finished based on our previous method[6]. The NOR test equipment contains an acrylic cube box (35 cm × 35 cm) with a white bottom. The test procedure is divided into three phases: habituation, familiarization, and test phase. On habituating day, each mouse was carefully placed into an empty box and given five minutes to explore the field. The area was cleaned with alcohol to clear the odor. On the day of training, two identical objects were located on the third point of the diagonal line of the box bottom. A mouse was placed in the center of the box and allowed to explore freely for 5 min. On the day of testing, one of the objects was substituted with a new one. The mice were placed in the box one by one and stayed for 5 min to recognize the two different objects, and their exploration track was recorded and analyzed by the tracking system.

Y maze. Y maze device is made of a black medical organic board with three arms of the same size and shape at 30 cm*8 cm*15 cm, and the angle between arms is 120°. The end of each wall is pasted with colorful figures with different shapes as a visual mark. Gently place the mouse into the middle area where the three arms meet and allow it to explore freely. Record the sequence of mice entering each arm within 8 min. Alternation is defined as consecutively entering three arms, such as 1, 2, 3; 1, 3, 2; 3, 2,

1 or 2, 1, 3. As long as the mouse enters three different arms continuously in a row, it can be regarded as an alternation.

The alternation= $the\ total\ number\ of\ arm\ entry - 2$

Statistical analysis. In the same experiment, the age and sex of mice were kept in the same conditions and randomly allocated to different experimental groups. GraphPad Prism 7.0 software was applied for the statistical analyses. The data represent the mean ± SEM or mean ± SD, as described in the figure legend. Differences between the two groups were examined using Student's t-test, and ANOVA with Tukey's multiple comparison tests for 3 or more groups. Significant differences were considered if $p < 0.05$.

References:

[1] J. L. Huang, G. Jiang, Q. X. Song, X. Gu, M. Hu, X. L. Wang, H. H. Song, L. P. Chen, Y. Y. Lin, D. Jiang, J. Chen, J. F. Feng, Y. M. Qiu, J. Y. Jiang, X. G. Jiang, H. Z. Chen, X. L. Gao, *NAT COMMUN* **2017**, *8*, 15144.

[2] Q. Song, M. Huang, L. Yao, X. Wang, X. Gu, J. Chen, J. Chen, J. Huang, Q. Hu, T. Kang, Z. Rong, H. Qi, G. Zheng, H. Chen, X. Gao, *ACS NANO* **2014**, *8*, 2345.

[3] X. Gu, Q. Song, Q. Zhang, M. Huang, M. Zheng, J. Chen, D. Wei, J. Chen, X. Wei, H. Chen, G. Zheng, X. Gao, *J CONTROL RELEASE* **2020**, *322*, 31.

[4] X. Wang, X. Gu, H. Song, Q. Song, X. Gao, Y. Lu, H. Chen, *ANAL CHIM ACTA* **2015**, *893*, 77.

[5] L. Yao, X. Gu, Q. Song, X. Wang, M. Huang, M. Hu, L. Hou, T. Kang, J. Chen, H. Chen, X. Gao, *J CONTROL RELEASE* **2016**, *226*, 1.

[6] Q. Zhang, Q. Song, X. Gu, M. Zheng, A. Wang, G. Jiang, M. Huang, H. Chen, Y. Qiu, B. Bo, S. Tong, R. Shao, B. Li, G. Wang, H. Wang, Y. Hu, H. Chen, X. Gao, *Adv Sci* **2021**, *8*, 2001918.

[7] X. Gao, L. Yao, Q. Song, L. Zhu, Z. Xia, H. Xia, X. Jiang, J. Chen, H. Chen, *BIOMATERIALS* **2011**, *32*, 8613.
